# Chip-Based Comparison of the Osteogenesis of Human Bone Marrow- and Adipose Tissue-Derived Mesenchymal Stem Cells under Mechanical Stimulation

**DOI:** 10.1371/journal.pone.0046689

**Published:** 2012-09-28

**Authors:** Sang-Hyug Park, Woo Young Sim, Byoung-Hyun Min, Sang Sik Yang, Ali Khademhosseini, David L. Kaplan

**Affiliations:** 1 Department of Biomedical Engineering, Tufts University, Medford, Massachusetts, United States of America; 2 Department of Biomedical Engineering, Jungwon University, Goesan-eup, Chungbuk, Korea; 3 Center for Biomedical Engineering, Department of Medicine, Brigham and Women's Hospital, Harvard Medical School, Boston, Massachusetts, United States of America; 4 Harvard-Massachusetts Institute of Technology Division of Health Sciences and Technology, Massachusetts Institute of Technology, Cambridge, Massachusetts, United States of America; 5 Department of Orthopeadic Surgery, Medical School, Ajou University, Youngtong-Gu, Suwon, Korea; 6 Department of Molecular Science and Technology, Ajou University, Youngtong-Gu, Suwon, Korea; 7 Department of Electrical and Computer Engineering, Ajou University, Youngtong-Gu, Suwon, Korea; 8 Wyss Institute for Biologically Inspired Engineering, Harvard University, Boston, Massachusetts, United States of America; Instituto Butantan, Brazil

## Abstract

Adipose tissue-derived stem cells (ASCs) are considered as an attractive stem cell source for tissue engineering and regenerative medicine. We compared human bone marrow-derived mesenchymal stem cells (hMSCs) and hASCs under dynamic hydraulic compression to evaluate and compare osteogenic abilities. A novel micro cell chip integrated with microvalves and microscale cell culture chambers separated from an air-pressure chamber was developed using microfabrication technology. The microscale chip enables the culture of two types of stem cells concurrently, where each is loaded into cell culture chambers and dynamic compressive stimulation is applied to the cells uniformly. Dynamic hydraulic compression (1 Hz, 1 psi) increased the production of osteogenic matrix components (bone sialoprotein, oateopontin, type I collagen) and integrin (CD11b and CD31) expression from both stem cell sources. Alkaline phosphatase and Alrizarin red staining were evident in the stimulated hMSCs, while the stimulated hASCs did not show significant increases in staining under the same stimulation conditions. Upon application of mechanical stimulus to the two types of stem cells, integrin (β1) and osteogenic gene markers were upregulated from both cell types. In conclusion, stimulated hMSCs and hASCs showed increased osteogenic gene expression compared to non-stimulated groups. The hMSCs were more sensitive to mechanical stimulation and more effective towards osteogenic differentiation than the hASCs under these modes of mechanical stimulation.

## Introduction

The stromal component of bone marrow is known to contain stem cell populations capable of differentiating into adipocytes, chondrocytes, myoblasts and osteoblasts. Despite their therapeutic potential in tissue engineering [1], utilization of bone marrow-derived mesenchymal stem cells (MSCs) is limited because acquisition methods can be painful, anesthesia is required and yields of MSCs cells are low. Alternative stem cell sources to substitute for MSCs, particularly where they overcome some of the above limitations, would be a positive step for tissue engineering and regenerative medicine. Adipose tissue-derived stem cells (ASCs) are considered as an alternative stem cell source. Adipose tissue is considered an abundant source of stem cells obtained by less invasive and painful methods, including lipoaspiration [2], [3]. ASCs also do not present ethical or immunologic problems [4]. These cells can self-renew to generate lost or damaged tissues and can differentiate into adipocytes, osteoblasts, myocytes, chondrocytes, endothelial cells, and cardiomyocytes [5]. hASCs have strong proliferation ability, and maintain phenotype and multi-differentiation potential [6].

Stem cells actively sense, adapt and respond to their surrounding microenvironment and interactively responding to external signals. Stem cell differentiation *in vivo* and *in vitro* can be regulated by a variety of signals, with growth factors, cytokines, and other regulatory molecules widely used in stem cell biology [1], [7]. It is also well known that mechanical stimuli regulate cells coupling to the environment. Cellular response to mechanical stimulation has been investigated and considered as an important role in the differentiation of stem cells [8]–[10]. Mechanical load aligns collagen fibers and tissue reorganization increases function. Thus, mechanical loading is important for maintaining the physiological and mechanical properties of mature bone, as well as other tissues [9]. Mechanical loading is a positive stimulus for bone formation [10] and is an essential factor in bone metabolism [11]. In recent years, various approaches to enhance and control the lineage specific differentiation of stem cells using mechanical stimuli have been developed and presented in macro- and microscale levels [9], [12]–[23].

In previous macroscale studies, dynamic fluid flow increased mineralized matrix deposition in 3D perfusion culture of marrow stromal cells [24] and mechanical strain promoted osteogenesis of human bone marrow-derived stem cell (hMSCs) *in vitro*, verified by the upregulation of osteogenic marker proteins like alkaline phosphatase (ALP), osteocalcin, osteopontin, and type I collagen [15], [25]. Cyclic compression also increased transcript levels of core binding factor A1 (Cbfa1/Runx2) which is a runt-like transcription factor essential for osteogenic differentiation in hMSCs [26]. Furthermore, hMSCs differentiation was enhanced by electromagnetic- and pneumatic-cyclic compressive stimuli in our previous studies [22], [23]. Human adipose tissue-derived stem cells (hASCs) also exhibited bone cell-like phenotype upon mechanical stimulation by pulsating fluid flow (5-Hz pulse with a mean shear stress of 0.6 Pa) [17]. In another case, hASCs had accelerated calcium deposition in response to continuous (10%, 1 Hz) and intermittent (10%, 1 Hz, 10s rest)-cyclic tensile strains [14].

Recently, microscale engineering has been increasingly used to mimic the cellular microenvironment with high spatiotemporal precision and to present cells with mechanical and biochemical signals [27]–[29]. These approaches were performed on a chip provide microenvironments that attempt to partially mimic human organs, such as blood vessels, muscles, airways, liver, brain, gut, kidney, and bones. For example, a lung-on-a-chip system was designed to mimic breathing by applying vacuum to side chambers, stretching porous membranes to stimulate cells seeded on the both sides of the membrane [28]. The microdevice replicates dynamic mechanical distortion of the alveolar-capillary interface for inflammatory and toxicology applications. In bone tissue engineering, various static and dynamic mechanical stimuli based on microfabrication technology have been tested with cultured stem cells or precursor cells for understanding osteogenic mechanisms and molecular pathways [30]–[33]. Micropatterns and structures giving rise to gradients of static mechanical stresses can also be used to pattern lineages (osteogensis in high stress areas and adipogenesis in low stress areas) of stem cells [30]. Osteoblasts on nanotexture under mechanical loading upregulated fibronectin and Cfba expression [31]. A continuous-perfusion microchip enhanced mouse osteoblastic cells in terms of ALP activity with shear stress [32]. A three-dimensional (3D) culture system with poly(ethylene glycol) hydrogel in multilayered polymeric microdevices, capable of simultaneously applying a range of cyclic, compressive mechanical forces to mouse MSC, was demonstrated [33]. This system has an advantage in conducting mechanically active experiments in 3D culture environments. However, the system requires many complex steps to form cell-loaded cylindrical hydrogels in the microdevice and ultraviolet (UV) exposure, which may decrease cell viability. In our previous studies, we also developed microscale platforms actuated by electromagnetic and pneumatic forces to provide cyclic compressive stimuli to cells, and demonstrated that hMSCs were enhanced in terms of chondrogenic and osteogenic differentiation [22], [23]. However, there are still limits in heat generation and the manual closing of the fluidic channels, which prompt the need to continue to improve the utility of such systems, as well as to expand the scope of applications, such as that explored here for stem cell comparative outcomes.

In the present study, a microscale stem cell chip was developed to culture stem cells loaded into separated micro chambers and to assess their comparative responses by dynamic compressive stimulation using a microchip. The osteogenic outcomes of hASCs were compared with hMSCs under the same mechanical stimulation which was assessed using this microscale stem cell chip system. The stem cell microchip was designed to culture the two different kinds of stem cells (hMSCs and hASCs) loaded into separated cell culture chambers, but to apply uniform dynamic compressive stimulation simultaneously. After exposure to mechanical stimulation, the ability of the hASCs towards osteogenic differentiation was assessed by histochemical and immunofluorescent staining, osteogenic related cluster of differentiation (CD) markers and gene expression, all in comparison to the hMSCs. The stem cell microchip developed in this research offers advantages, including those that are generic (i.e. minimizing size, cost, and usage of materials) for microscale systems, as well as new features such as the concentric design of holes and cell chambers for uniform mechanical stimulation, embedded microvalve systems to improve convenience and minimize manual intervention in closing fluidic channels, and compartmentally paired cell culture chambers for collecting statistically relevant data from two different cell types in single experiments.

## Results

### Histochemical staining for osteogenesis

**Figure 1:**
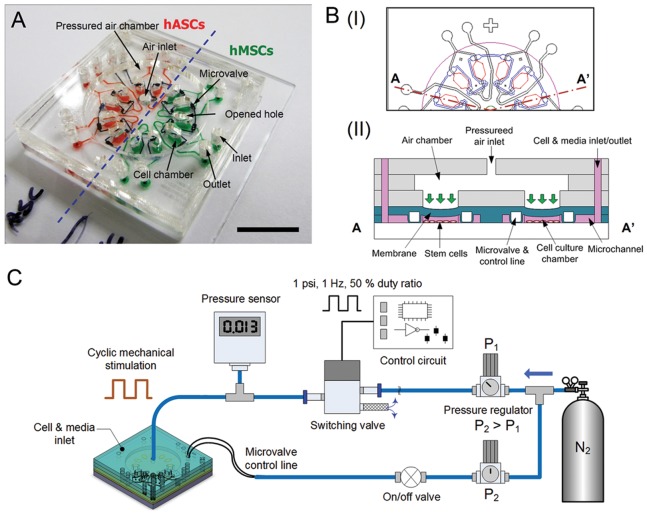
Microchip and experimental setup for evaluating stem cells towards osteogenesis under mechanical stimulation. (A) The microchip is comprised of a cover, an air chamber, looped microvalves, and twelve cell culture chambers. These paired cell chambers share the inlet/outlet channel. The cells (hMSCs and hASCs) are loaded into half of the chip, individually. Scale bar = 1 cm. (B) Schematic diagram of top view (I) and simplified cross-sectional view (II) of the device. The device was designed to culture two different stem cells simultaneously and to apply mechanical stimulation using cyclic pneumatic force. (C) The experimental setup for mechanical stimulation, including a controlled nitrogen gas pressurized air chamber. The frequency of pneumatic pressure is controlled with a switching solenoid valve derived by a control circuit. During mechanical stimulation, microvalves are closed with higher pressure (P2>P1) to prevent undesired shear stress in the cell chambers.

**Figure 2:**
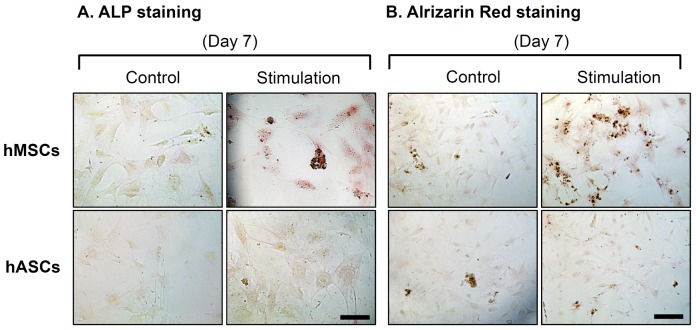
Osteogenesis characterizations of hMSCs and hASCs after 7 days. hASCs and hMSCs cultured in the microchip with osteogenic medium for 7 days were stained with ALP and Alrizarin red. The stimulated group of BMSCs resulted in significantly enhanced ALP activity and calcium deposits. (Scale bars: ALP staining 100 (m, Alrizarin red staining 200 (m).

The new stem cell microchip bioreactor was designed and fabricated (**Fig. 1**) and then used to assess cellular responses. ALP was assessed by histochemical analysis as a marker of the commitment towards an osteoblastic lineage and correlated with advanced matrix mineralization and mature phenotype. hMSCs were more densely stained in the mechanical stimulation groups compared to the nonstimulated group, while stimulated hASCs did not show a significant rise in ALP staining compared to nonstimulated hASCs **(** **Fig. 2A** **)**. Alizarin red staining is based on the capacity of alizarin red to specifically stain matrix containing calcium and its positive appearance is considered an expression of bone matrix deposition. This staining showed enhanced calcium deposition in the stimulated groups of hMSCs at day 7. hASCs did not show any difference between stimulated and nonstimulated groups **(** **Fig. 2B** **)**.

### Immunofluorescent staining for osteogenic markers and cell surface markers

**Figure 3:**
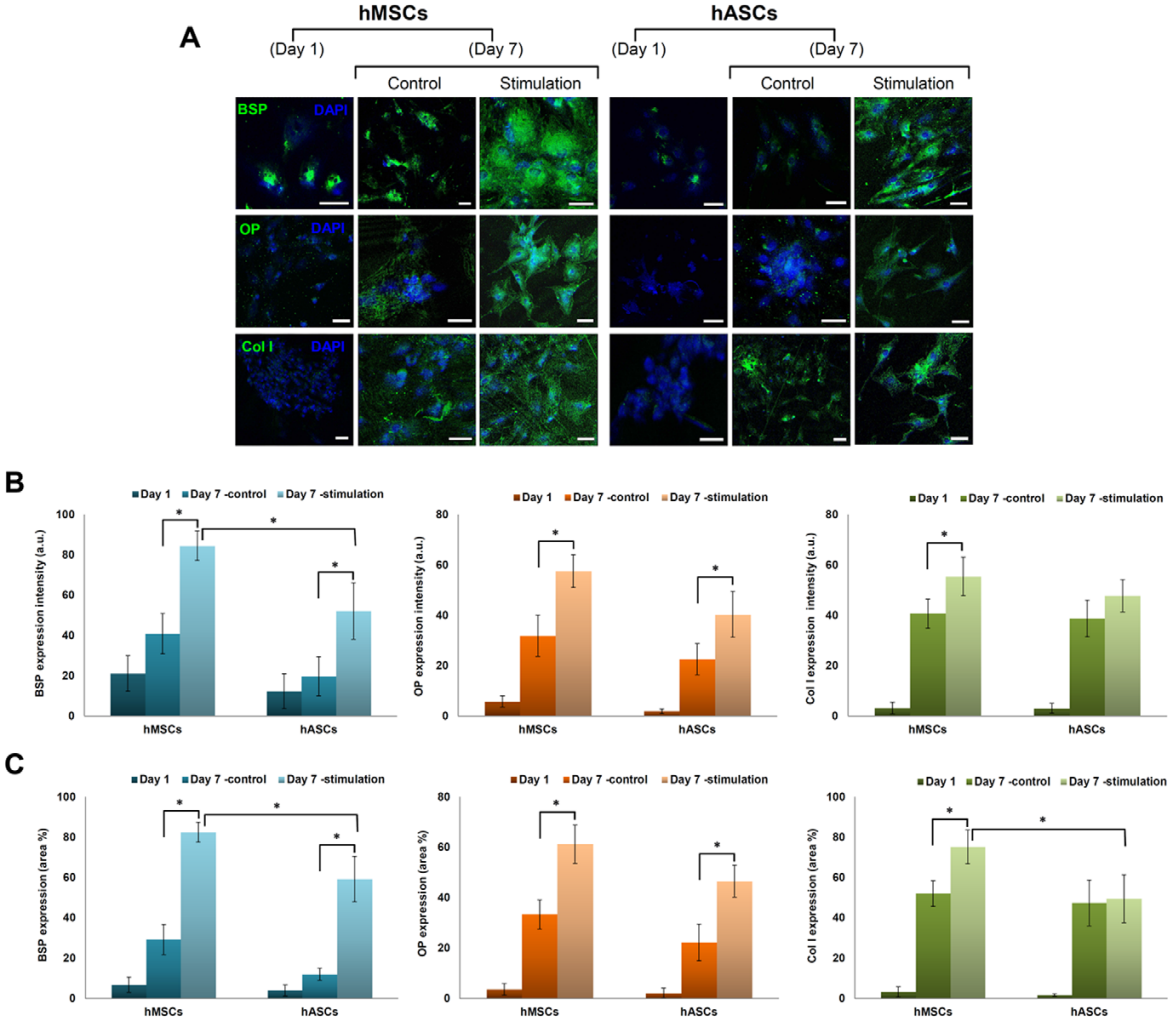
Immunocytochemical staining of hMSCs and hACSs. (A) The expression of osteogenic markers after 1 and 7 days. Bone sialoprotein (BSP), Osteopotin (OP), and Collagen type I (Col I) were stained with GFP and strongly expressed in the stimulated BMSCs. Blue = DAPI Nucleic Acid Stain. (Scale bars: 100 (m) Green fluruorecent expression intensity (B) and area (C) of ECMs in hMSCs and hASCS. Data presented in the line graph represent mean value with SD (n = 12). *p<0.05. Star (*) indicates comparison of statistical difference of stimulation to control and statistical difference between stimulated hMSCs and hASCs.

Immunocytochemistry confocal images were taken to examine expression of BSP, OP (representative proteoglycans in osteogenesis) and Col I (representative collagen type in osteogenesis) after 7 days **(** **Fig. 3A** **)**. Although the expression of these components increased with time for both stem cell types, the features of ECM deposition by stimulation were different. Mechanical stimulation resulted in an increase in the area and intensity of BSP in the hMSCs (*p<0.05), and hASCs (*p<0.05). In particular, the expression area and intensity of BSP in the hMSCs was qualitatively higher than in the hASCs under the same mechanical stimulation (*p<0.05). OP was also increased in the stimulated groups of both stem cells compared to nonstimulated groups (*p<0.05). Type I collagen expression also significantly increased depending on mechanical stimulation in the hMSCs, while it did not increased in the hASCs. This indicates that synthesis of ECMs was influenced by mechanical stimulation which affected the deposition density of BSP, OP and type I collagen **(** **Fig. 3B and 3C** **)**.

**Figure 4:**
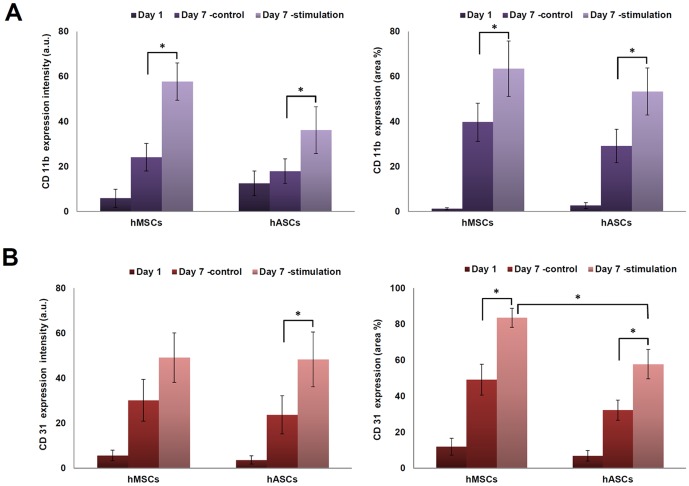
Expressions of integrin CD11b and CD31. (A) Fluorescent expression intensity and area of CD11b in hMSCs and hASCS. (B) Green fluorescent expression intensity and area of CD31 in hMSCs and hASCS. Star (*) indicates comparison of statistical difference of stimulation to control in the same cell type. *p<0.05. It also indicates statistical difference between stimulated hMSCs and hASCs.

External stress caused by mechanical stimulation is known to change CD markers of cells. To examine the changes of cell surface receptors of the two stem cell types, CD31 (PE-CAM) and CD11b of β2 integrin were evaluated **(** **Fig 4A** **and Fig S1)**. CD11b was expressed at low levels at day 1 in both stem cell types. At day 7, the expression of CD11b did not differ significantly in cells cultured in the non-stimulated controls. However, changes were observed for both cell sources cultured under stimulation **(** **Fig 4A** **)**. **Fig. S2** shows the expression of CD31, which was elevated when hMSCs were stimulated. Stimulated hASCs also showed significantly higher expression of CD31 compared to controls (*p<0.05). However, expression of CD31 in the hMSCs was statistically higher compared to in the hASCs (*p<0.05) **(** **Fig. 4B** **)**. After 7 days under stimulation, confocal images of actin were obtained to investigate cytoskeleton organization. The results showed that stained actin filaments were denser in the stimulated hMSCs and hASCs compared to the nonstimulated groups **(Fig. S1 and S2)**.

### Osteogenic gene expression

**Figure 5:**
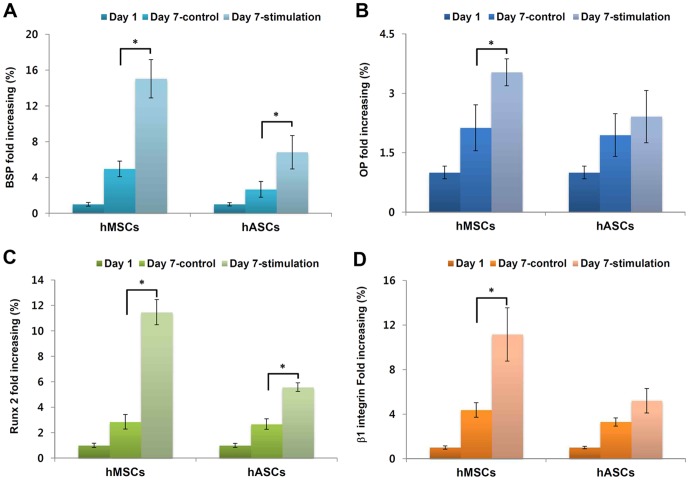
Osteogenesis related transcript levels and β1 integrin expression. (A) Bone sialoprotein (BSP), (B) Osteopontin (OP), (C) Runt-related transcription factor 2 (Runx2), (D) β1 integrin (*p<0.05)

Transcript levels of osteogenic markers such as BSP, OP, Runx-2 and β1 integrin were analyzed by real-time PCR (Brilliant II, Stratagene, USA) **(** **Fig. 5** **)**. After 7 days, transcript levels of all genes increased in both stem cell groups compared to day 1 levels. In particular, mechanically stimulated stem cells resulted in increased expression compared to the nonstimulated stem cells. Comparing hMSCs and hASCs, expression of BSP in stimulated hMSCs and hASCs was 3- and 2-fold higher than those in nonstimulated cell, respectively **(** **Fig. 5A** **)**. OP transcription levels in stimulated hMSCs were 1.7 times higher than nonstimulated control. However, hASCs did not show a statistical difference in control after 1 week **(** **Fig. 5B** **)**. The result of Runx-2 expression indicated that stimulated hMSCs and hASCs increased transcript level around 4- and 2-fold, respectively **(** **Fig. 5C** **)**. For β1-integrin transcripts, the expression level of the stimulated hMSCs was 2.4 higher than nonstimulated hMSCs. In contrast, hASCs did not show statistically different in β1-integrin expression levels between the stimulated and nonstimulated groups **(** **Fig. 5D** **)**.

## Discussion

The importance of mechanical stimulation in the regulation of stem cell differentiation has been identified, thus increasing the need for efficient systems to perform mechanical stimulation on cells in a rapid and controllable manner. The physiological mechanisms by which bone and progenitor cells sense mechanical forces *in vivo* can be better understood through *in vitro* experimentation where mechanics is included. Recently, diverse approaches have been performed to enhance and control chondrogenic and osteogenic differentiation of stem cells using mechanical stimuli. Various systems have been developed to provide a certain range of mechanical stimuli such as the direct movement of integrins, deformation of the substrate by stretching or bending, steady or oscillatory fluid flow, hydrostatic pressure, and hypergravity [34]. The most widely used systems for mechanical stimulating are bending or stretching systems, such as four-point bending devices and Bioflex® culture systems (Flexcell International Corp., USA) [9], [12]–[21]. Mechanical cyclic uniaxial tensile strain (0.5 Hz) may induce the differentiation of MSCs into osteoblasts with increased ALP activity and upregulated mRNA levels of Cbfa1 and ALP, which is vital for bone formation in distraction osteogenesis [18]. However, those macroscale systems require a large number of cells, large space for cell culture, and a significant volume of expensive medium and biochemical materials for histochemical and immunocytochemical analysis. Most current macroscale stimulators and conventional products also have an open structure, which has the possibility of contamination and requires a clean environment during the stimulation. Therefore, there is a demand for miniaturized systems to minimize cost, contamination risk, and labor and external equipment needs.

Previously, we developed microscale stimulation systems actuated by electromagnetic and pneumatic forces for studying osteogenesis of rabbit and human MSCs under mechanical stimulation [22], [23]. The microscale cell exciter used electromagnetic actuators to deliver cyclic-compressive loads to rabbit MSCs in 3D disk-shaped alginate gels [22]. The results were promising in demonstrating that mechanical stimulation enhanced the synthesis of cartilage-specific matrix proteins and markers. However, there were also some limitations. Heat and electromagnetic field (EMF) generated from electromagnetic coils can disturb experimental results since these factors can influence protein synthesis and fate outcomes in stem cells. Additionally, handling problems exists in this system related to contamination due to the open structure. The pneumatic microchip has an improved design compared to the earlier electromagnetic device. To eliminate electromagnetic fields and heat generation problems, we adopted pneumatic force as the actuating source. The experimental results using the pneumatic device exhibited similar data to support that mechanical compression can accelerate the osteogenic differentiation of hMSCs [23].

hMSCs and hASCs have shown promise as a source of expandable and pluipotent cells for tissue engineering and regenerative medicine [35]. They may be stimulated with different types of mechanical inputs based on the state of differentiation and source. In particular, hASCs require additional verification of functions, including a more complete understanding regarding optimal *in vitro* culture conditions to generate functional engineered tissues. Even though mechanical stimuli play an important role in osteogenesis of hMSCs and hASCs, most comparative studies of MSCs and ASCs have focused on *in vitro* differentiation using cytokines or *in vivo* implantation for osteogenic evaluation [36]. Just few studies have focused on the response of ASCs to mechanical stimulation in comparative assessments to the osteogenic outcomes of MSCs. Comparative data of MSCs and ASCs on the osteogenic capacity under mechanical stimulation are crucially required because key in the context of musculoskeletal tissue is the *in vitro* generation of nascent tissue with appropriate mechanical stimuli. To investigate the potential ability of hASCs towared osteogenic differentiation under mechanical stimulation, new systems are required in order to provide mechanical stimulation to different kinds of stem cells simultaneously, as well as to allow for the separate culture of the stem cells in different cell chambers. Therefore we designed a novel microscale device able to culture different types of stem cells (hMSCs and hASCs) and applied uniform hydraulic compressive pressure to cells in this study. Even though previous pneumatic devices also have multiple cell chambers, it is challenging to apply the same magnitude of stimulation pressure on each chamber [22]. To provide uniform pneumatic forces on each cell chamber, all cell chambers were located along a concentric center with same distance from the inlet port, which is connected to the pneumatic pressure source. In the electromagnetic cell stimulator described earlier, cell chambers had an open-structure and individual electromagnetic actuators were built under each cell chamber [23]. Therefore, the applied mechanical stimulation based on the electromagnetic attraction force had some variations, which depended on the thickness of alginate gel placed between the bottom of cell chamber and the metal cap. The other type of cell chambers in the pneumatic chip was designed to have different lengths from the air inlet port for applying various amplitudes of pressure from one pressure source. To eliminate the limitations of previous systems in uniform stimulation, the novel system described here has concentric-located cell culture chambers as described earlier. This design provides uniform pressure distribution on multiple cell chambers inside the device for testing comparative mechanical stimulation on different types of stem cells simultaneously.

The embedded structure adopted in this microchip also has an advantage in minimizing external contamination. In addition, an on-chip control microvalve system is integrated to minimize unexpected shear stress inside the cell chamber during stimulation by closing the inlet and outlet channels of the cell chamber with relatively high pressure. In the previous experiment using the pneumatic chip, we manually clamped silicone tubes connected to each inlet/outlet ports with locking forceps. This was time consuming and labor-intensive, as well as tricky, because it can expose the cultures to contaminants. The on-chip control microvalve can close multiple inlet and outlet channels simultaneously by applying pressure into the valve control line. The embedded microvalve system is more efficient, convenient, time-saving and safe than the manual method.

The molecular mechanisms responsible for the adaptation of connective tissue to mechanical loading is clinically relevant, especially for bone, but also for other mechanically sensitive tissues [37]. Cells bind to matrix proteins via several different types of adhesion receptors including integrins. Integrins are a major family of heteodimeric receptors that span the cell membrane, linking matrix components on the outside of the cells to cytoskeletal, adaptor, and signaling molecules on the inside of the cell [38]. The role of integrins in cell motility is closely related to adhesive functions, which is relevant for both osteoblast precursors and osteoclasts [34]. Proliferation and progressive differentiation of MSCs, osteoprogenitors, and osteoblasts in culture are associated with changes in the types and expression levels of integrin and matrix ligands and activation of integrin signaling. Bidirectional integrin signaling is important for dynamic cell processes in bone such as adhesion, proliferation, differentiation, and potentially also mechanotransduction [37].

The integrins are composed of noncovalently linked α and β subunits. We analyzed the expression of adherence molecules using CD31 (PE-CAM), CD11b of β2 integrin after exposure to mechanical stimulation. CD11b/CD18 (Mac-1, αmβ2, mo1, CR3) receptors can recognize a wide range of structurally unrelated ligands and transfer the information from the outside to the inside of the cell [39], [40]. CD11b/CD18 receptors promote cellular adhesion and like most transmembrane receptors are capable of transmitting signals elicited by ligand binding, resulting in cellular effector responses [40], [41]. CD11b is a characteristic integrin that is important in cell adhesion and phosphorylation activation events mediated through tyrosine kinase and phosphatidyl inositol 3 kinase [40]. Expression of CD11b with CD18 (with subfamily, αmβ2) on BMSCs significantly enhances bone formation *in vivo*, whereas genetic inactivation of CD18 in mice leads to defective osteogenesis due to decreased expression of the osteogenic master regulator Runx2/Cbfa1 [42]. CD11b/CD18 is also essential for osteogenic differentiation [43].

CD31 is a cell-adhesion molecule involved in the amplification of integrin-mediated cell adhesion, maintenance of the adherent junction integrity, organization of the intermediate filament cytoskeleton, regulation of transcriptional activities, and control of apoptotic events [44]. CD31 facilitates the interaction of osteoprogenitors with other cells, such as endothelial cells, by homophilic interactions between CD31 on various cells or and the heterophilic interaction between CD31 and integrin [45]. Osteosarcoma cells were shown to express αvβ3 integrin, which has been found to be a ligand for CD31. In addition, metastasis of osteosarcoma cells to other bones was significantly correlated with expression of bone morphogenetic protein (BMP) and CD31 [45]. The expressions of CD31 of hMSCs and hASCs were increased by mechanical stimulation compare to controls at day 7 in the present study (Fig 4).

Furthermore, we confirmed elevated β1 integrin gene expression by mechanical stimulation (Fig. 5D). β1 integrins play an important role in osteoblast differentiation as well as in bone remodeling [34]. Recent studies demonstrated essential roles for integrins, particularly the β1, β2, and β3 subfamilies, in bone formation and remodeling. Upon application of a mechanical stimulus to bone cells, both β1 integrins are redistributed in the plane of the membrane and proteins associated with focal adhesions are phosphorylated [20]. In addition, mechanical stimulation of osteoblast lineage cells can increase production of integrins, ECM proteins, and growth factors, often in an integrin-ECM dependent manner. Increased expression of β1 integrin and matricellular protein, such as OP, is commonly upregulated in response to mechanical stimulation both *in vitro* and *in vivo* [21]. The application of strain *in vitro* to human osteosarcoma cells selectively upregulates mRNA for β1 integrin [12], and steady fluid shear up regulates expression of integrin β1 in normal human osteoblasts [16]. Direct distortion of β1 integrin in osteoblast lineage cells causes increased focal adhesion formation, phosphorylation of tyrosine kinase [46], and localized waves of intercellular calcium release [47]. These results provide evidence that integrins on osteoblasts and osteocytes have the ability to detect a mechanical load and translate the physical stimulus into a chemical response.

ALP is a cell surface glycoprotein that is involved with mineralization [48]. ALP expression showed an increase in activity with mechanical stimulation in hMSCs compare to hASCs in the present study. Apart from ALP, OP and BSP showed an increase in staining intensity with stimulation (Fig. 3).

Osteogenic genes (BSP, OP and Runx 2) were examined with real-time PCR after 7 days. Cbfa1/Runx2 and Protein C-ets-1 (Ets-1) are transcription factors, which play important roles in regulating the expression of a wide variety of genes responsible for the osteoblast phenotype [18], [49]. Runx2 binds to osteoblast-specific cis-acting element 2, which is located in the promoter region of osteocalcin gene. Expression of osteoblast phenotype-related genes such as OC, type I collagen, ALP, BSP, OP, and collagenase-3 is down-regulated in the absence of Runx2 [50]. OP is expressed throughout matrix maturation, followed first by BSP [51]. In addition, secreted OP and BSP participate in matrix formation and they can bind cell surface integrin receptors and regulate mineralization [19]. In previous studies, a cyclic uniaxial tensile strain (0.5 Hz, 2000 microstrain) promoted MSCs proliferation, increase ALP activity and up-regulate the expression of Cbfa1 and Ets-1. A significant increase in Ets-1 expression was detected immediately after mechanical stimulation but Cbfa1 expression was elevated later. [18], [52] In this study, BSP, OP and Runx2 in both stimulated groups (hMSC and hASC) increased at day 7, In particular, stimulated hMSCs showed statistically higher expression than stimulated hASCs. The results presented suggest that hMSCs were more sensitive and responsive to cyclic compressive mechanical stimulation compared to hASCs under the conditions studied here.

The feasibility of using this pneumatically actuated microscale chip was demonstrated as a convenient and effective tool for comparative stem cell studies responsive to mechanical stimulation. The chip reduces the quantity of stem cells required for screening, reduces process costs and time, and increases throughput for various stimulation conditions. In addition, the device has many advantages compared to the previous systems, such as concentric-located holes on each cell chamber for the uniform stimulation, embedded microvalve system for improving convenience and minimizing contamination, and compartmentalized cell chambers for the culture of different types of cells for collecting reliable and statistical data in two different cell types. Mechanical stimuli affect many different physical and biochemical phenomena at the cellular level, including proliferation and biosynthetic activity. With the knowledge gained through this type of bioreactor system and study, new options to understand mechanotransduction and cellular responses to mechanical stimulation can be developed and used to investigate optimal conditions for osteogenesis for bone tissue engineering and regenerative medicine needs.

## Conclusions

hMSCs exposed to mechanical stimulation showed distinct ALP and Alrizarin outcomes, while hASCs did not show positive staining under the same experimental conditions. Dynamic compressive mechanical stimulation (1 Hz, 1 psi) increased osteogenic ECM formation (BSP, OP, Col I) and integrin (CD11b and CD31) levels in both stem cell types (hMSCs and hASCs). Upon application of mechanical stimulation to the two types of stem cells, integrin (β1) and osteogenic gene transcripts were upregulated. The results demonstrated that hMSCs were more sensitive to mechanical stimulation compared to hASCs. The microchip presented here, which has embedded concentric-located holes on each cell chamber and a microvalve system, was demonstrated in terms of utility for comparative stem cell studies in response to mechanical stimulation. Further studies are needed to identify the primary osteogenic signals associated with cyclic compressive mechanical stimulation and to determine the mechanism by which these influence commitment to and progression through the osteogenic lineage. By selectively applying specific mechanical stimuli *in vitro*, it may be possible to determine the most effective range of conditions to stimulate osteogenesis of human stem and progenitor cells.

## Materials and Methods

### Design and fabrication of the stem cell microchip

The stem cell microchip was designed to be able to apply uniform dynamic compressive stimulation to hMSCs and hASCs generated by a pulsatile pneumatic pressure. A photograph and schematics (top and cross-sectional views) of the system are shown in **Figs. 1A and 1B**. The stem cell microchip consists of a radial shaped pneumatic actuator with a flexible membrane and the array of cell culture chambers. To provide a uniform mechanical stimulation to the stem cells, six paired cell culture chambers are located along a concentric circle of the centered air inlet **(** **Fig. 1A** **)**. These cell chambers can be filled with the different types of stem cells to assess responses under the same mechanical stimulation. Each cell chambers can be visualized with green and red dyes (green: hMSCs, red: hASCs) **(** **Fig. 1A** **)**. The microdevice is operated based on a pneumatic actuator with a flexible polymer diaphragm. There is one air chamber, six paired cell culture chambers and an embedded microvalve system (**Fig. 1B** **(I)**). During the mechanical stimulation period, the stimulating pressure generated from the regulated nitrogen gas is applied to a PDMS membrane that transmits to the media and cell membrane. Simultaneously, the integrated microvalve system was actuated to close all inlet and outlet channels connected to each cell chamber to minimizing undesired shear stress in the cell chambers (**Fig. 1B** **(II)**). This can be attained when applying pressure (5 psi) that was five times greater than the stimulating pressure (1 psi) for the valve control. The air inlet was connected to the pulsatile pressurized air (e.g. nitrogen gas), which was controlled with a fast switching solenoid valve **(** **Fig. 1C** **)**.

The device had two main components: one consisting of three poly(methylmethacrylate) (PMMA) substrates and the other with two poly(dimethylsiloxane) (PDMS) layers and one glass substrate. The dimensions of the device were 30 mm×30 mm×10 mm. Both PDMS and PMMA are biocompatible and transparent, so that the cell cultures can be observed with a microscope. The PMMA components, including the cover with air inlet, plate for the air chamber and hole-plate were prepared with a computer controlled laser-cutting machine (VersaLASER, USA). The cover has one air inlet (green circle) at the center, two access holes for the valve control line (blue line) and twelve inlets and outlets (black circles) to access the cell culture chambers (**Fig. 1B** **(I)**). The PMMA plate for the air chamber works as a gasket with one big hole in the center (pink circle). The hole-plate has twelve windows (red line) with the same sized cell culture chambers to allow the desired pressure to be attained with each PDMS membrane. The microvalve and cell culture chambers are made by a standard molding-process using thick-negative photoresist (SU-8, MicroChem, USA) mold [23]. SU-8 master molds for the microvalve and cell culture chambers have different heights (150 µm and 200 µm) on silicon wafers. To reduce the cost of the mold process, inexpensive material (e.g. polyethylene glycol diacrylate (PEGDA)) with photoinitiator could be used for rapid molding instead of the more expensive materials used in the present version [53]. Mixed PDMS solutions (prepolymer∶curing agent = 1∶10) were degassed over 2 hours in a vacuum chamber, poured onto the master molds, and cured at 80°C for 2 hours in an oven. Cured PDMS layers were detached from the molds and punched to make inlets and outlets for fluidic connections. The surface of the PDMS layers was activated with oxygen plasma (Plasma cleaner, Harrick Plasma, USA) and bonded to the glass substrate. The volume of one pair of cell culture chambers and channels is 1.32 µL and 16.78 µL, respectively. The total volume to fill the entire space of the device, which includes six pairs of cell culture chambers and channels, is approximately 150 µL. The surface of three PMMA substrates are treated with chloroform (Sigma, USA) and bonded with each other. A layer of silane radicals was formed on the bottom surface of the bonded PMMA part using dilute 3-aminopropyl triethoxysilane (3-APTES) after oxygen plasma treatment [54]. Finally, the surface treated PMMA and plasma treated PDMS layers were bonded for the device fabrication. The PDMS substrate was placed between the 2 mm-thick PMMA and glass substrates to minimize deformation of PDMS during stimulating experiments. Fabricated chips were sterilized with ethylene oxide (EO) gas for 24 hours. To remove toxic residues after sterilization, the chips were kept in the vacuum oven for a minimum of 72 hours under vacuum.

### hMSC and hASC culture

hMSCs were isolated and expanded using our previously published protocols [55]. Human bone marrow aspirates (25 ml, Lonza, 27 year-old male, Walkersville, Inc., MD) were diluted in 75 ml of (1x) phosphate buffered saline (PBS). The cells were separated by density gradient centrifugation. Twenty ml aliquots of bone marrow suspension were overlaid onto a poly-sucrose gradient (1077 g/cm^3^, Histopaque, Sigma, St. Louis, MO) and centrifuged at 800×g for 30 min at room temperature. The cell pellet was resuspended in Minimum Essential Medium Eagle (α-MEM: Gibco BRL, Grand Island, NY) supplemented with 10% fetal bovine serum (FBS, Gibco BRL), 100 U/mL penicillin G (Gibco BRL) and 100 µg/mL streptomycin (Gibco BRL).

hASCs were obtained from a 30 year-old female donor abdomen lipoaspirate (Pennington Biomedical Research Center, Baton Rouge, USA). The hASCs were expanded from collagenase-digested stromal vascular fraction cells in stromal medium consisting of DMEM/F12 Ham's medium, 10% FBS, 100 U/mL penicillin G and 100 µg/mL streptomycin. Cell number and viability were determined using trypan blue exclusion. The resuspended cells were plated at a density of 1.5×10^5^ cells/cm^2^ and placed in a 5% CO_2_ incubator at 37°C. The culture medium was changed every other day. Passage two cells were dissociated with 0.25% trypsin–EDTA at 80% confluency before being used for experiments.

### Mechanical stimulation

To investigate the osteogenic differentiation potential ability of hASCs compared to hMSCs under the dynamic mechanical stimulation, hASCs and hMSCs were separately mixed with medium (density: 2.5×10^6^ cells/mL), and manually loaded into each cell culture chamber through microchannels (width: 300 µm, height: 200 µm) with a 1 mL plastic syringe (BD Medical, USA). The loaded stem cell chips were placed in a humidified incubator (5% CO_2_, 37°C) overnight. To apply the hydraulic compressive pressure to the stem cells (hMSCs and hASCs), the chips were connected to the pneumatic control setup describe in **Fig. 1C**. The pneumatic control setup consists of two precision pressure regulators (LRP series, FESTO, Germany), a fast switching solenoid valve (MHE2 series, FESTO, Germany), a driving circuit, a pressure gauge, an on/off valve, and pneumatic tubes. Nitrogen gas pressure was controlled with two precision pressure regulators with different pressure levels (P_1_ = 1 psi, P_2_ = 5 psi). The pressure (P_2_) for the microvalve control was set higher than the stimulating pressure (P_1_) to maximize closing efficiency of the microvalve. To generate dynamic compressive pressure for the mechanical stimulation, the regulated pressure was controlled with a fast switching solenoid valve driven by electric circuit with pulsatile signal (frequency: 1 Hz, duty ratio: 50%). The pulsatile pressure was applied into the air chamber through the air inlet of the cover. During the stimulation period, the microvalve was activated to close all inlets and outlets of the cell culture chambers to prevent fluid flow. The cultured hASCs and hMSCs on the bottom surfaces of cell culture chambers were periodically exposed to the mechanical stimulation for 10 min every 12 hours for 7 days. After exposure, the microchips were kept in a humidified incubator for the duration of experiment. The control group was treated with the same procedure except for the application of the mechanical stimulation. Osteogenic media consisted of α-MEM for hMSCs and DMEM/F12 for hASC supplemented with 10% FBS, 0.1 mM nonessential amino acids, 50 µg/mL ascorbic acid-2-phosphate, 100 nM dexamethasone and 10 mM β-glycerolphosphate in the presence of 100 U/ml penicillin, 100 µg/ml streptomycin, and 0.25 µg/mL fungizone. Each cell culture chamber was supplied with fresh osteogenic medium daily. During experiments, each inlet and outlet of micro chamber was connected to a pair of 20 µL-pipet tips filled with different volume of fresh media to maintain the continuous medium supply and prevent air bubble formation in the microchips. This is a passive-supply method based on the differential head of media, which minimizes shear stress during the supply of fresh media. This approach allows cells in the microfluidic device to survive for reasonably long-term (7 days) without the medium drying out or experiencing nutrient deficiency.

### Histochemical staining

To analyze the osteogenic differentiation of stem cells in the microchips, ALP was assessed by histochemical analysis using staining kit (Sigma). Alkaline assay mixture was prepared with the standard recipe (2.4 mg fast violet B salt (Sigma) and 0.4 mL naphthol AS-MX phosphate alkaline solution (Sigma) in 9.6 mL of distilled water). Cells in all culture chambers were incubated in a dark room for 45 min with the alkaline-mixture by injecting solution (over 250 µL in each device) into chambers. For alizarin red staining, cells were fixed in 4% formaldehyde after washing twice with PBS. The cells were stained with 40 mM alizarin red S (pH 4.2, Sigma) for 10 min. All stained cells were observed with a Leica DMIL light microscope (Watzlar, Germany) and Leica Application Suite (v3.1.0) software after washing twice with PBS.

### Immunofluorescent staining

To stain for cell response, cell culture medium was gently removed and cell culture chambers were gently washed twice with PBS (pH 7.4). Subsequently, the samples were fixed with 4% paraformaldehyde solution for 10 min at room temperature. The 4% paraformaldehyde was removed with three PBS washes. The cells were then permeabilized with PBS (pH 7.4) containing 0.2% Triton X-100 for 10 min, and blocked with PBS (pH 7.4) containing 1% bovine serum albumin (BSA) for 30 min. After dilution, the solution was placed onto each sample for 30 min with two subsequent PBS rinses. Primary antibodies for type I collagen (rabbit, Abcam, Inc., Cambridge, MA), bone sialoprotein (BSP) (rabbit, Abcam, Inc., Cambridge, MA), and osteopontin (OP) (rabbit, Abcam, Inc., Cambridge, MA) were diluted from their respective stock solutions to 5–10 µg/mL concentrations in PBS. Then 250 µL of antibody solution was placed into each chamber on devices and incubated at 4°C for 3 hours. The samples were then washed 3 times with PBS and stained using fluorescein isothiocyanate (FITC) (anti-rabbit, Abcam, Inc., Cambridge, MA) as secondary antibody, in which a 10 µg/mL dilution was prepared. A 250 µL aliquot of secondary antibody solution was added into each chamber for 1 hour with two subsequent PBS rinses.

The changes in surface markers on the stimulated stem cells were examined by immunofluorescence staining on cells in a monolayer using FITC-conjugated anti-human monoclonal antibodies, CD11b (Thy-1, Abcam) and CD31 (PECAM1, Abcam). Cells were fixed for 5 min in 4% paraformaldehyde and washed twice with PBS. FITC-monoclonal anti-CD11b and CD31 were applied for assessment of the expression of proteins. After diluting, washing twice with PBS for 10 min each time, actin filaments were stained using Texas Red-X phalloidin stain (Invitrogen, Inc., Grand Island, NY), which was diluted using 10 µL of methanol stock reagent and 400 µL of PBS for each sample.

Confocal microscopy was carried out to examine cytoskeleton and extracellular matrix (ECM) structures. The middle z-section images of cells were taken using a Leica TCS SP2 AOBS confocal microscope (Leica, Mannheim, Germany) equipped with 488 nm argon and 543 nm He/Ne lasers. Phalloidin staining was excited at 543 nm and emission collected between 580 and 650 nm. FITC secondary antibody excitations were at 488 nm, and emission collected between 500 and 550 nm. Image J software (Ver. 1.44P, NIH) was used to quantify the mean fluorescent intensity and the area (%) occupied by positive staining, following immunohistology of osteogenic ECMs and CD markers. [56], [57] Each gray scale image for green fluorescent staining was separated from the RGB channels and normalized to remove background staining. To measure the mean background fluorescence intensity for each slide, two boxes were placed in background areas in which there was no binding by primary antibody. For the analysis, 12 images were captured from three different chambers were used for statistical analysis.

### Real-time quantitative polymerase chain reaction (Q-PCR)

Total RNAs from each specimen were extracted using Trizol reagent (Invitrogen, Carlsbad, CA) and Micro RNeasy Micro kit (Qiagen, Hilden, Germany). hMSCs and hASCs culture chambers were filled with Trizol. All detached cells were collected into 1.5 mL tubes after 30 min. Chloroform (100 µL) was added to the solution and incubated for 5 min at room temperature. Tubes were again centrifuged at 12,000 g for 15 min and the upper aqueous phase was transferred to a new tube. All samples were homogenized by vortexing for 1 min after adding 20 ng carrier RNA (5 µL of a 4 ng/µL solution). Continuously, one volume of 70% ethanol (v/v) was added and applied to an RNeasy minElute spin column.

The RNA samples were reverse transcribed into cDNA using oligo (dT)-selection according to the manufacturer's protocol (High Capacity cDNA Archive Kit, Applied Biosystems, Foster City, CA). Runx 2, BSP and OP levels were quantified using the Mx3000 Quantitative Real Time PCR system (Stratagene, La Jolla, CA) for osteogenesis and β1-integrin for a cell surface marker. All data analysis employed the Mx3500 software (Stratagene) based on fluorescence intensity values after normalization with an internal reference dye and baseline correction. Differences of gene expression were generated by a using comparative Ct method (Ct [delta][delta] Ct comparison). Ct values for samples were normalized to the endogenous housekeeping gene. PCR reaction conditions were 2 min at 50°C, 10 min at 95°C, and then 50 cycles at 95°C for 15 s, and 1 min at 60°C. The data were normalized to the expression of the housekeeping gene, glyceraldehyde-3-phosphate-dehydrogenase (GAPDH) within the linear range of amplification and differences [58]. The GAPDH probe was labeled at the 5′ end with fluorescent dye VIC and with the quencher dye TAMRA at the 3′ end. Primer sequences for the human GAPDH gene were: forward primer 5′-ATG GGG AAG GTG AAG GTC G-3′, reverse primer 5′-TAA AAG CCC TGG TGA CC-3′, probe 5′-CGC CCA ATA CGA CCA AAT CCG TTG AC-3′. Probes for Runx-2, BSP, OP and β1-integrin were purchased from Assay on Demand (Applied Biosciences, Foster City, CA).

### Statistical analysis

Statistical difference in biochemical and mechanical quantitative analysis were determined using the Mann-Whitney U test (Independent t-test, SPSS). Statistical significance was assigned as *p<0.05.

## Supporting Information

Figure S1 **Immunocytochemical staining of integrin CD11b and actin.** Green staining indicates the immunostained CD 11b, Red staining indicates the immunostained actin phalloidin, Overlay images of CD11b and actin phalloidin. (Scale bars: 100 µm).
(TIF)

Figure S2 **Immunocytochemical staining of integrin CD31 and actin.** Green staining indicates the immunostained CD 11b, Red staining indicates the immunostained actin phalloidin, Overlay images of CD31 and actin phalloidin. (Scale bars: 100 µm).
(TIF)
