# Correction: Chip-Based Comparison of the Osteogenesis of Human Bone Marrow- and Adipose Tissue-Derived Mesenchymal Stem Cells under Mechanical Stimulation

**DOI:** 10.1371/journal.pone.0334482

**Published:** 2025-10-22

**Authors:** Sang-Hyug Park, Woo Young Sim, Byoung-Hyun Min, Sang Sik Yang, Ali Khademhosseini, David L. Kaplan

After publication of this article [1], concerns were raised about results presented in Fig 2. Specifically, the ALP staining hMSCs Control panel appears to partially overlap with the Alrizarin Red staining hMSCs Control panel.

The first author stated that the ALP staining hMSCs Control panel in Fig 2 is incorrect. A corrected version of Fig 2 is provided here where the ALP staining hMSCs Control panel has been updated, the Alrizarin Red label and caption of [1] have been corrected to Alizarin Red, and the scale bars units have been updated to µm. The underlying data for Fig 2 is also provided here in S1-S5 Files.

The first author stated that the remainder of the original raw data supporting the figures in the article are available.

A member of the *PLOS One* Editorial Board reviewed the underlying data and updated version of Fig 2 and stated that other data in [1] corroborate what Fig 2 intends to demonstrate.

**Fig 2:**
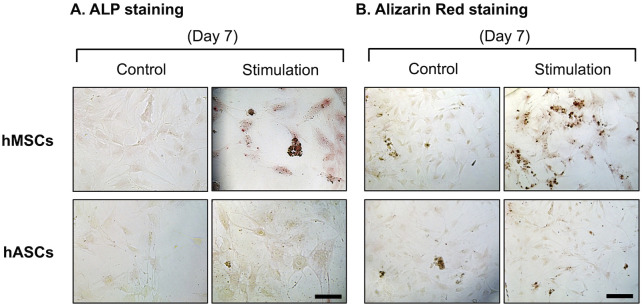
Osteogenesis characterizations of hMSCs and hASCs after 7 days. hASCs and hMSCs cultured in the microchip with osteogenic medium for 7 days were stained with ALP and Alizarin red. The stimulated group of BMSCs resulted in significantly enhanced ALP activity and calcium deposits. (Scale bars: ALP staining 100 (µm), Alizarin red staining 200 (µm)).

## Supporting information

S1 File The original raw data supporting the hMSCs Control Alizarin red stained panels in the corrected Fig 2 and replication data from the time of the original experiments. (ZIP)

S2 File The original raw data supporting the hASCs Control Alizarin red stained panels in the corrected Fig 2 and replication data from the time of the original experiments. (ZIP)

S3 File The original raw data supporting the Stimulated Alizarin red stained panels in the corrected Fig 2 and replication data from the time of the original experiments. (ZIP)

S4 File The original raw data supporting the Control ALP stained panels in the corrected Fig 2 and replication data from the time of the original experiments. (ZIP)

S5 File The original raw data supporting the Stimulated ALP stained panels in the corrected Fig 2 and replication data from the time of the original experiments. (ZIP)

## References

[1] Park S-H, Sim WY, Min B-H, Yang SS, Khademhosseini A, Kaplan DL. Chip-based comparison of the osteogenesis of human bone marrow- and adipose tissue-derived mesenchymal stem cells under mechanical stimulation. PLoS One. 2012;7(9):e46689. doi: 10.1371/journal.pone.0046689 23029565 PMC3460891

