# Supplementary figures and images for: Chip-Based Comparison of the Osteogenesis of Human Bone Marrow- and Adipose Tissue-Derived Mesenchymal Stem Cells under Mechanical Stimulation
Source: PLoS One. 2012 Sep 28;7(9):e46689. doi: 10.1371/journal.pone.0046689 (PMC3460891; doi:10.1371/journal.pone.0046689)

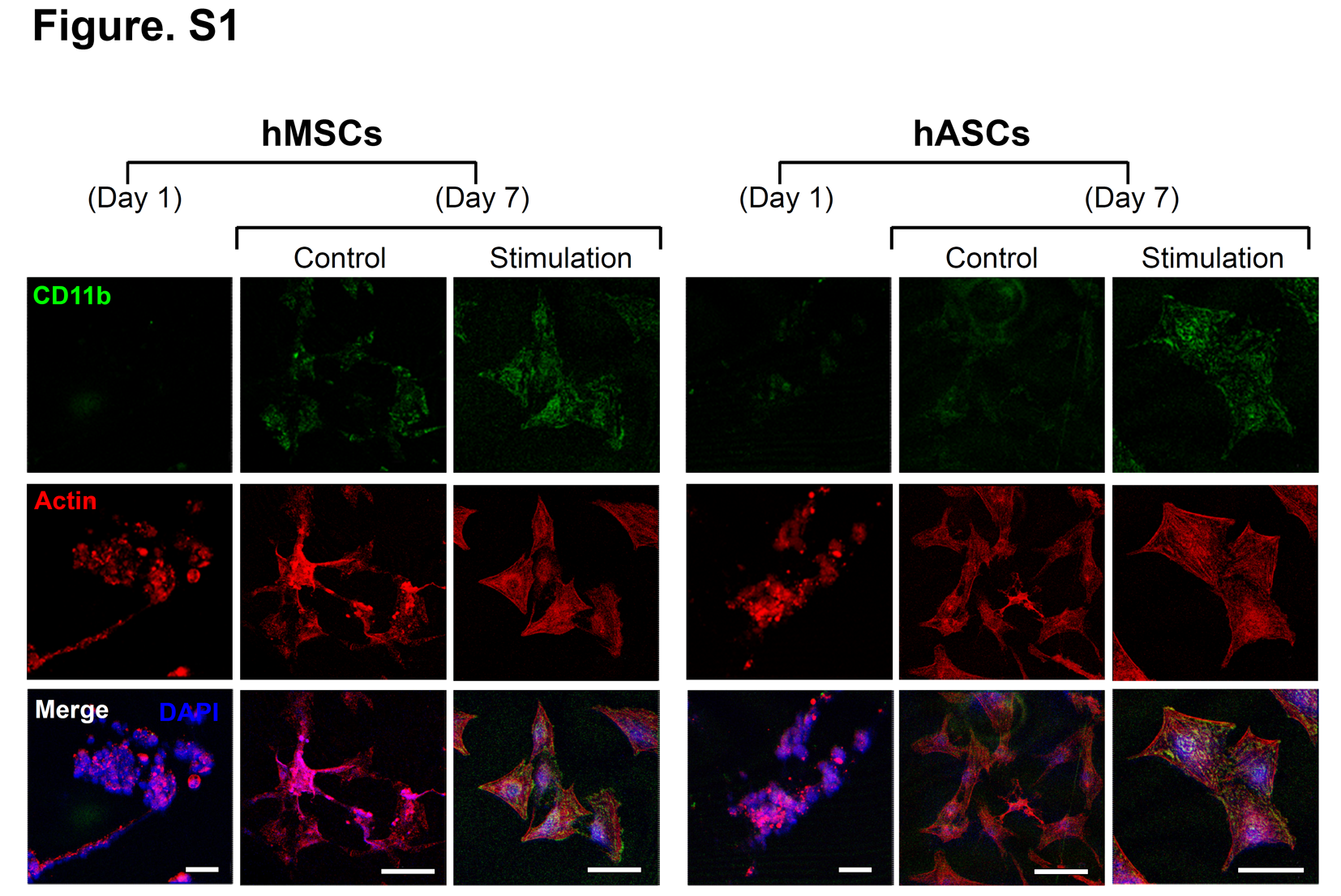

Supplement: Figure S1 — Immunocytochemical staining of integrin CD11b and actin. Green staining indicates the immunostained CD 11b, Red staining indicates the immunostained actin phalloidin, Overlay images of CD11b and actin phalloidin. (Scale bars: 100 µm). (TIF) [file pone.0046689.s001.tif]

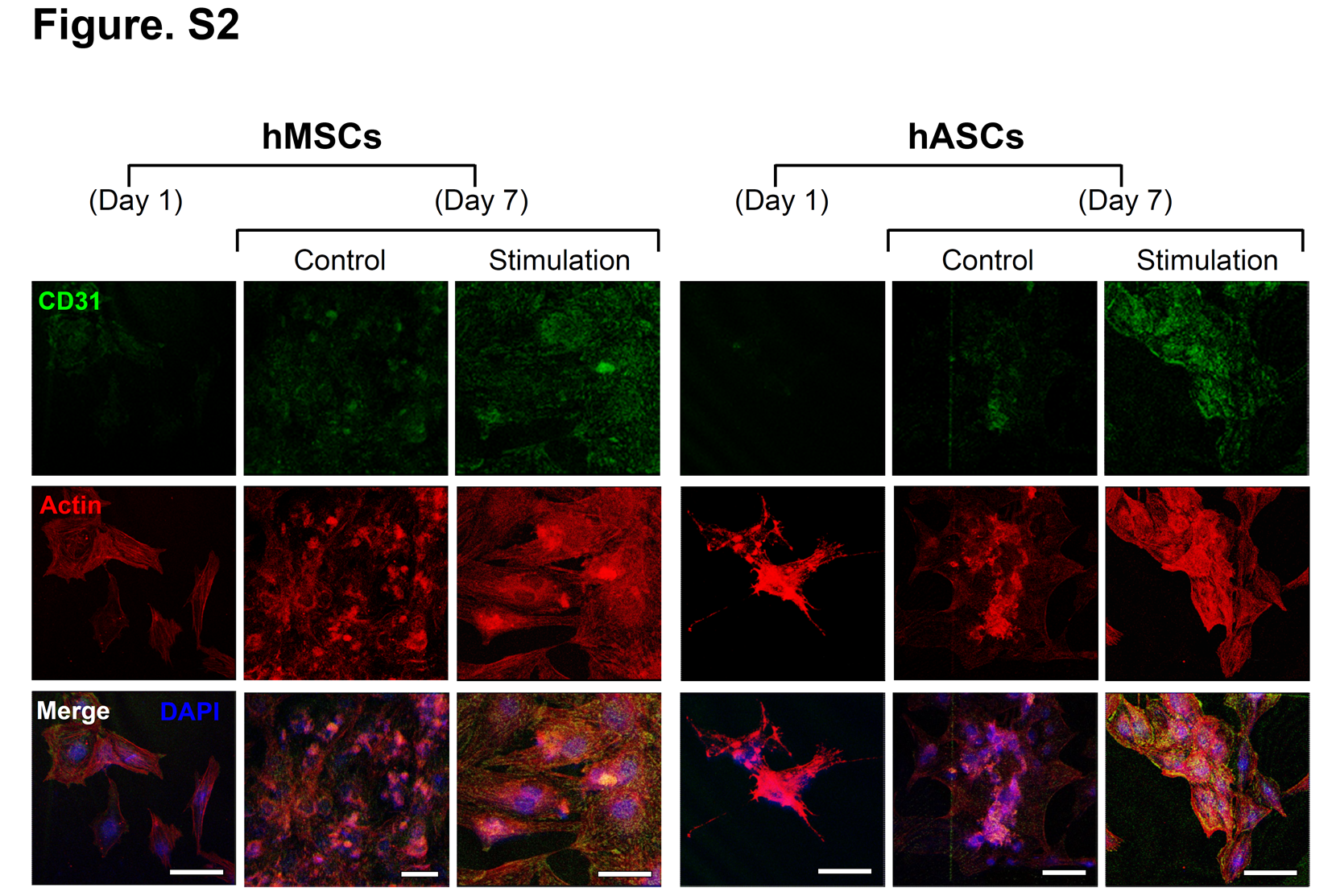

Supplement: Figure S2 — Immunocytochemical staining of integrin CD31 and actin. Green staining indicates the immunostained CD 11b, Red staining indicates the immunostained actin phalloidin, Overlay images of CD31 and actin phalloidin. (Scale bars: 100 µm). (TIF) [file pone.0046689.s002.tif]
