# Supplementary figures and images for: Correction: Chip-Based Comparison of the Osteogenesis of Human Bone Marrow- and Adipose Tissue-Derived Mesenchymal Stem Cells under Mechanical Stimulation
Source: PLoS One. 2025 Oct 22;20(10):e0334482. doi: 10.1371/journal.pone.0334482 (PMC12543147; doi:10.1371/journal.pone.0334482)

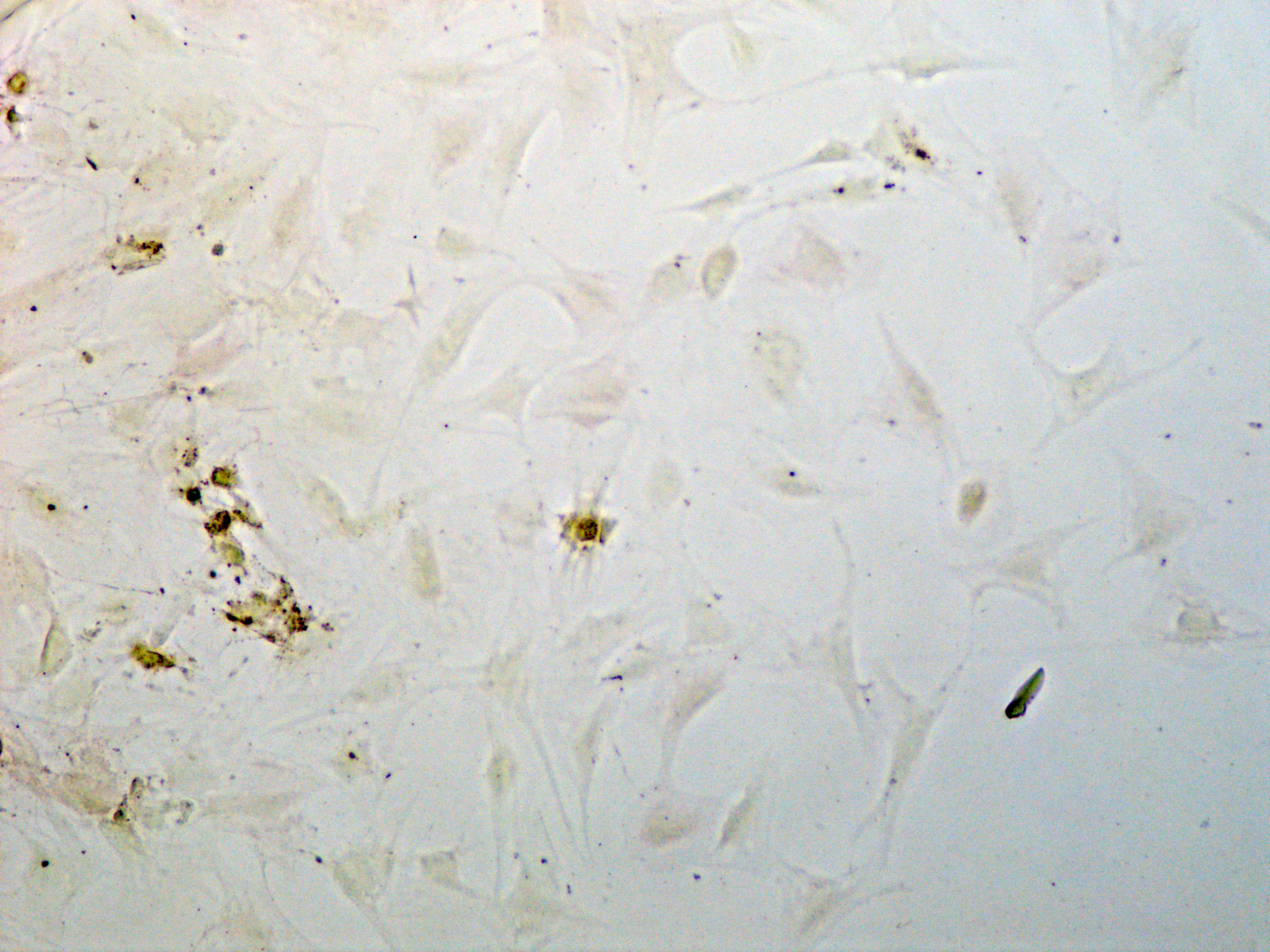

Supplement: S1 File — (ZIP) [file pone.0334482.s001.zip › 12-AR 100-day 7-NS-MSC2.tif]

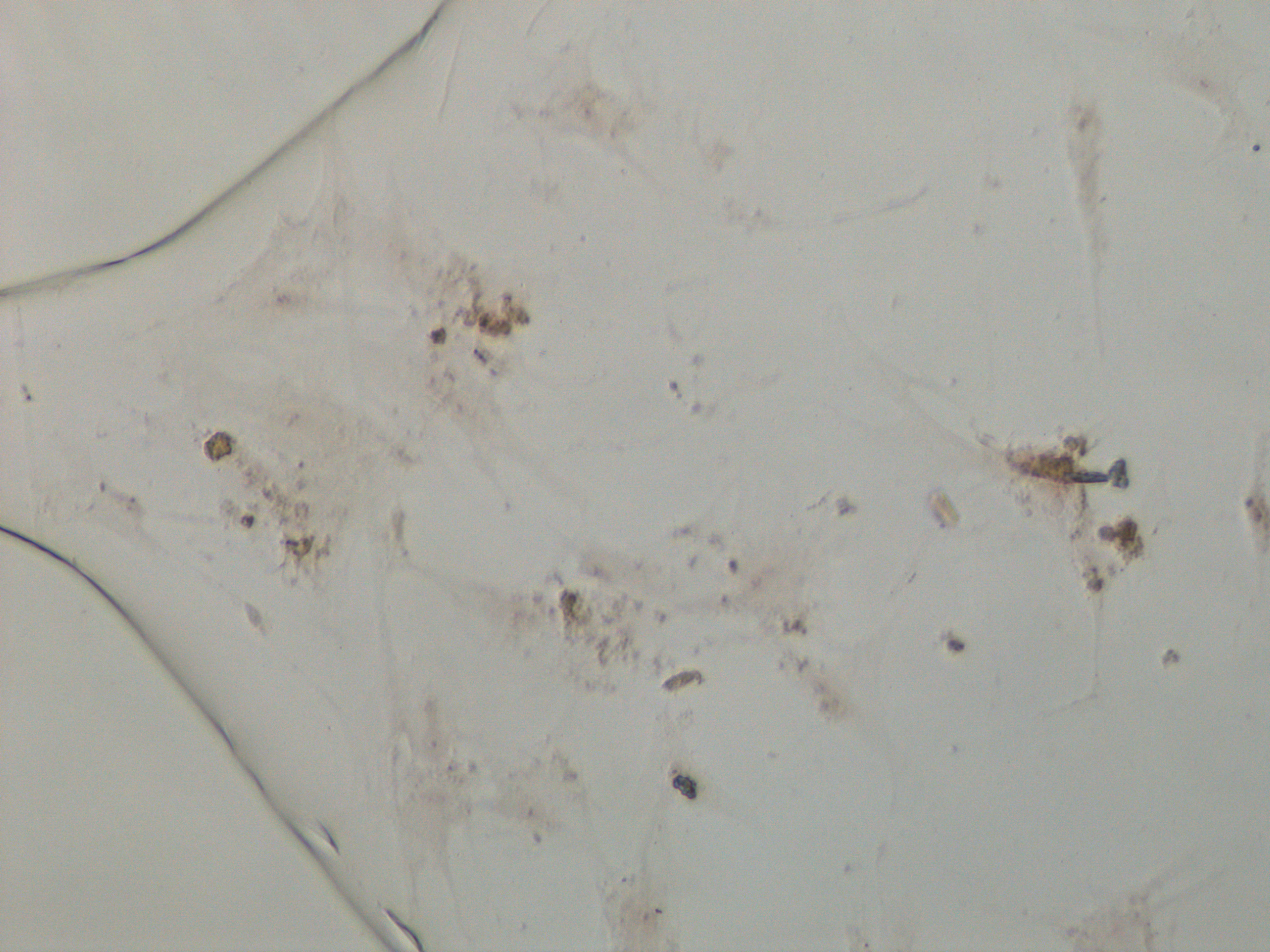

Supplement: S1 File — (ZIP) [file pone.0334482.s001.zip › Alrizarin 100-day 7-NS-MSC6.tif]

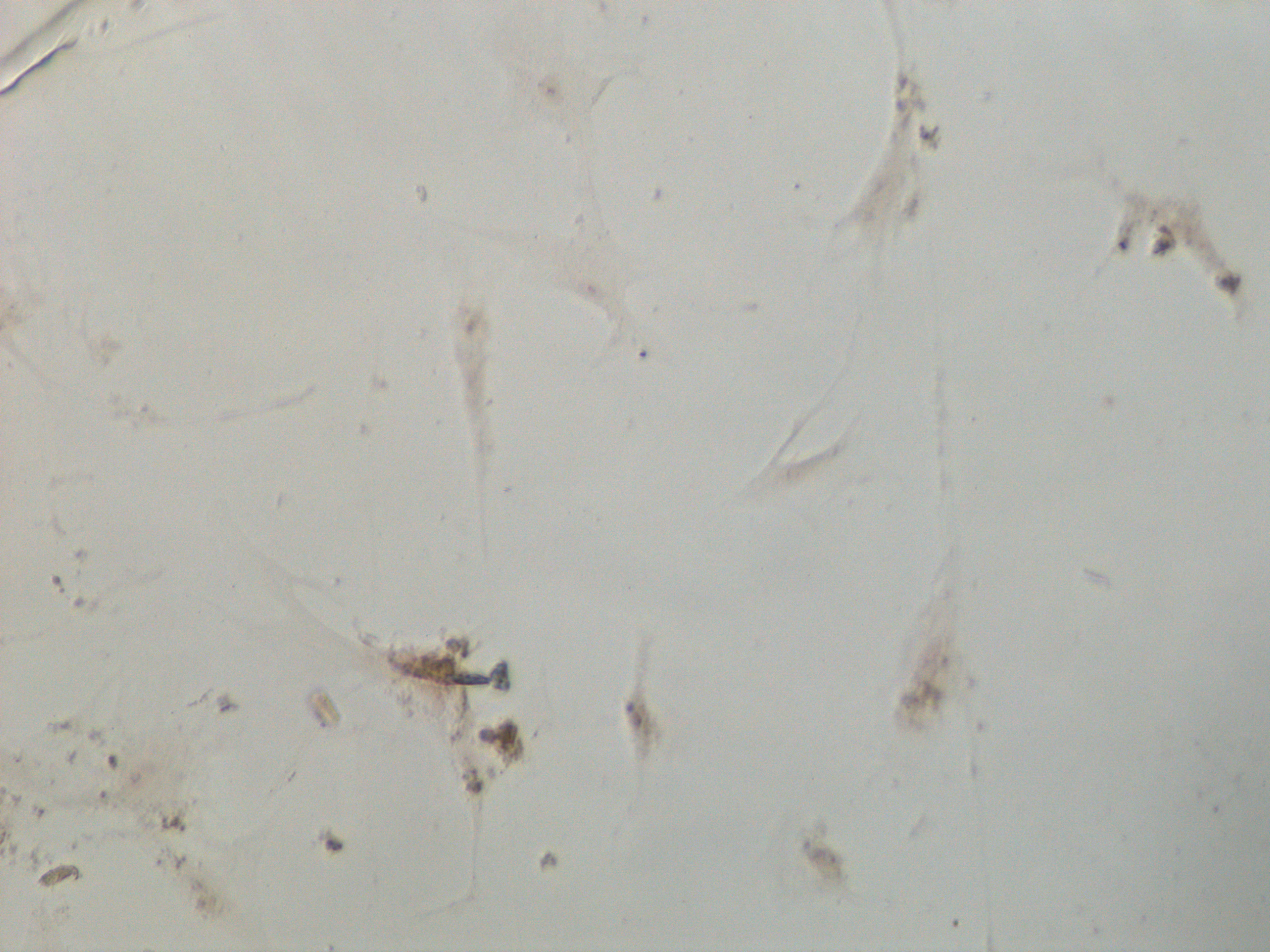

Supplement: S1 File — (ZIP) [file pone.0334482.s001.zip › Alrizarin 100-day 7-NS-MSC7.tif]

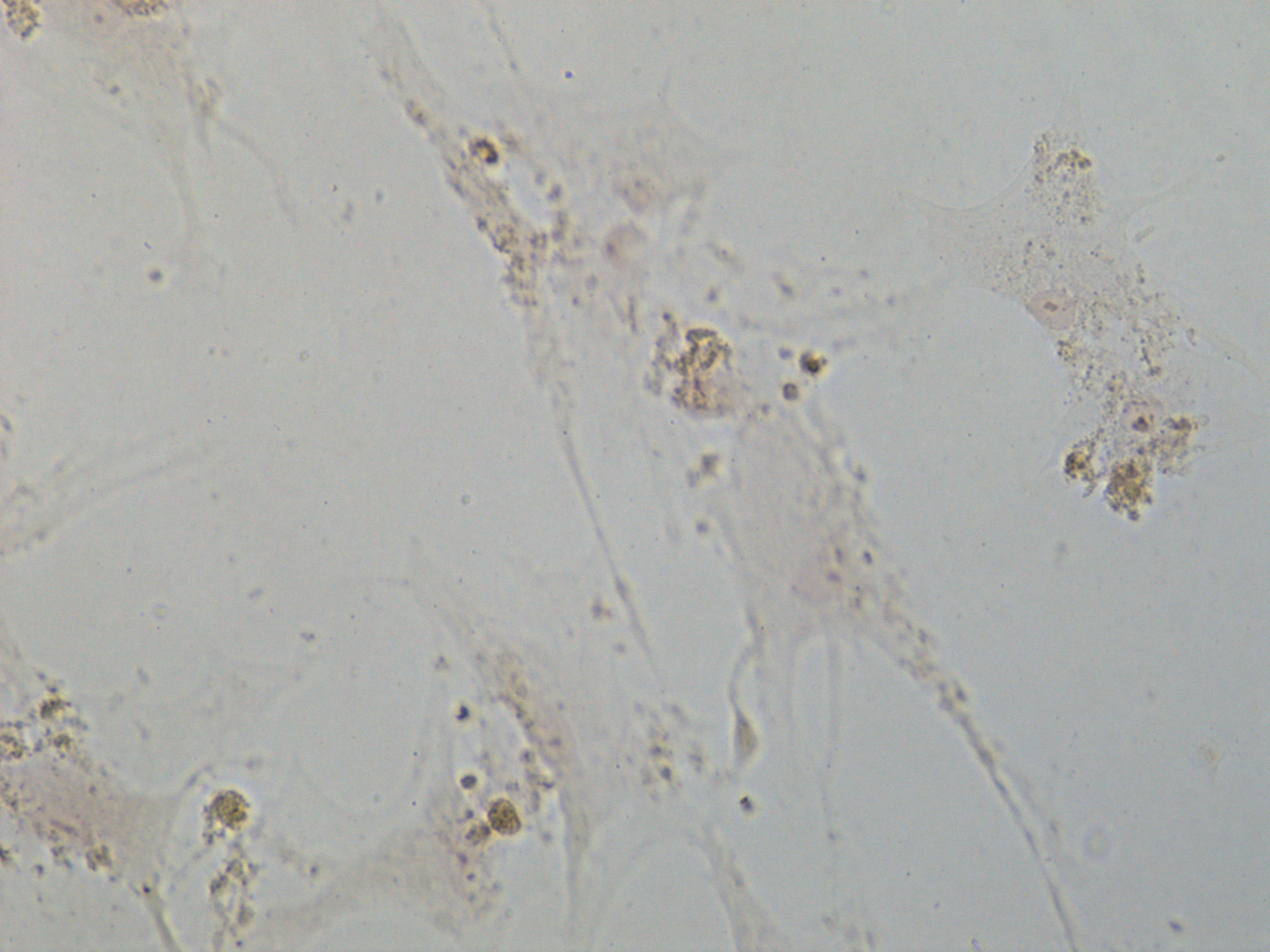

Supplement: S1 File — (ZIP) [file pone.0334482.s001.zip › Alrizarin 200-day 7-NS-MSC5.tif]

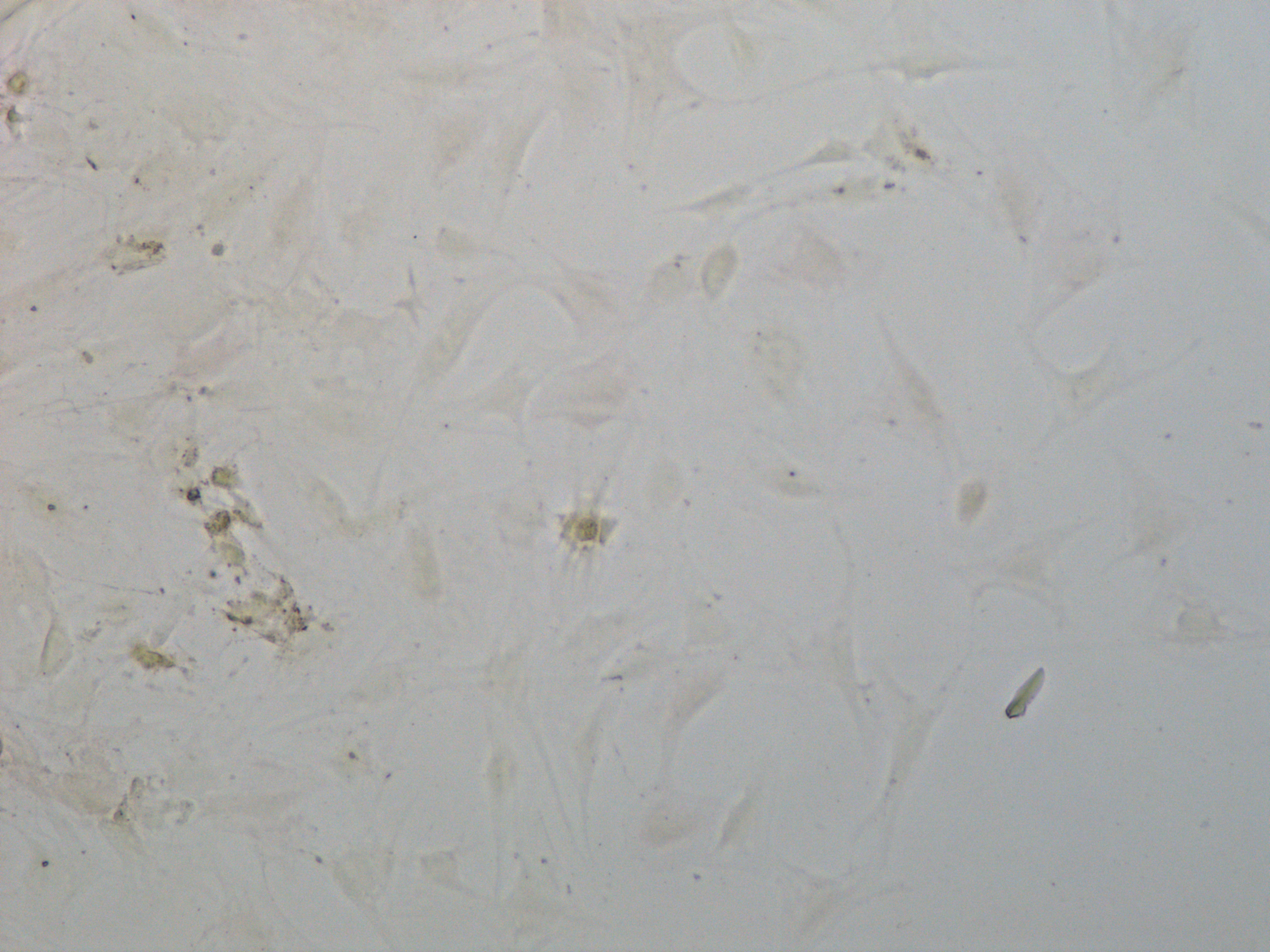

Supplement: S1 File — (ZIP) [file pone.0334482.s001.zip › AR 100-day 7-NS-MSC2.tif]

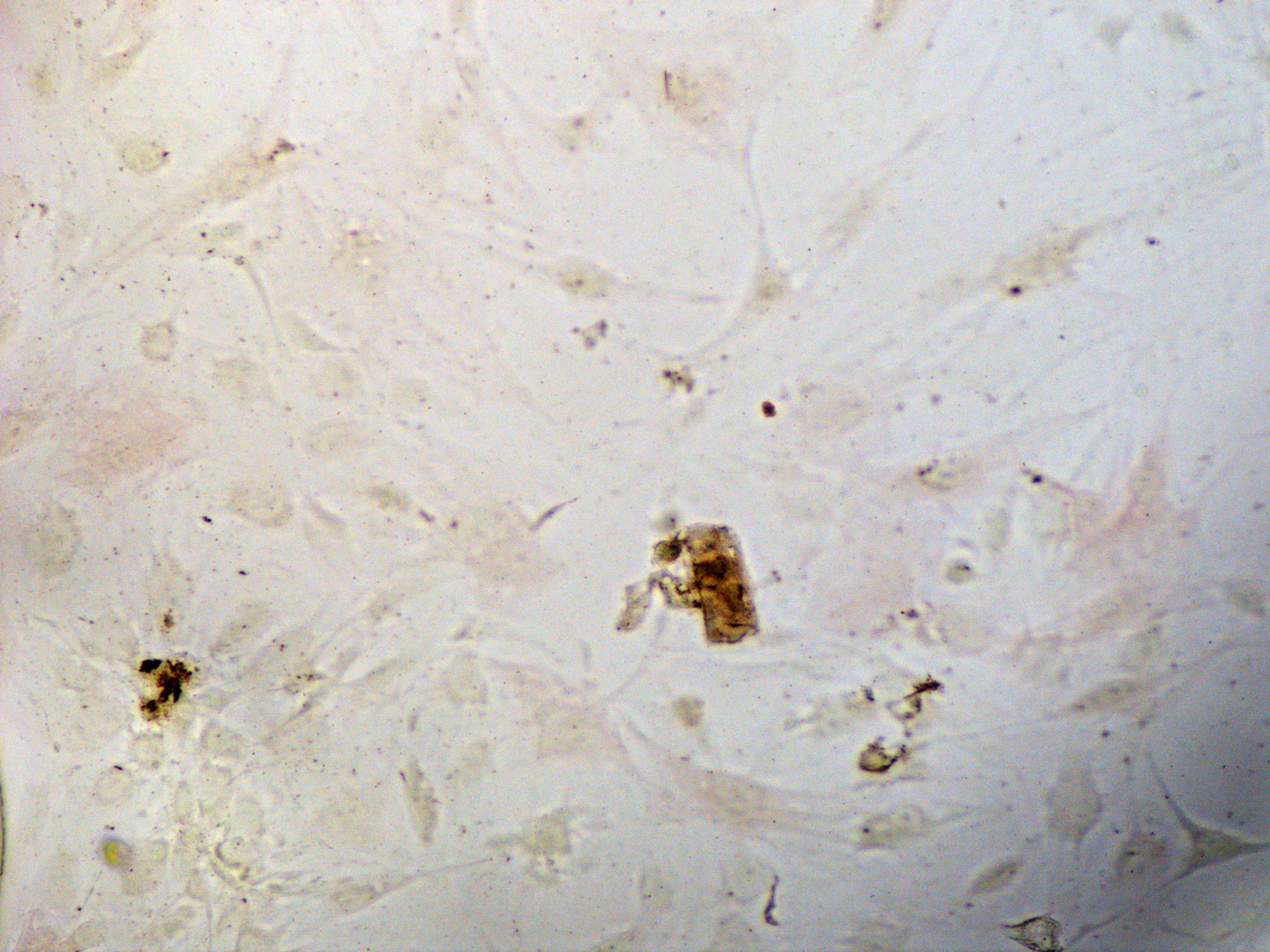

Supplement: S2 File — (ZIP) [file pone.0334482.s002.zip › 11-AR100-day 7-NS-ASC6.tif]

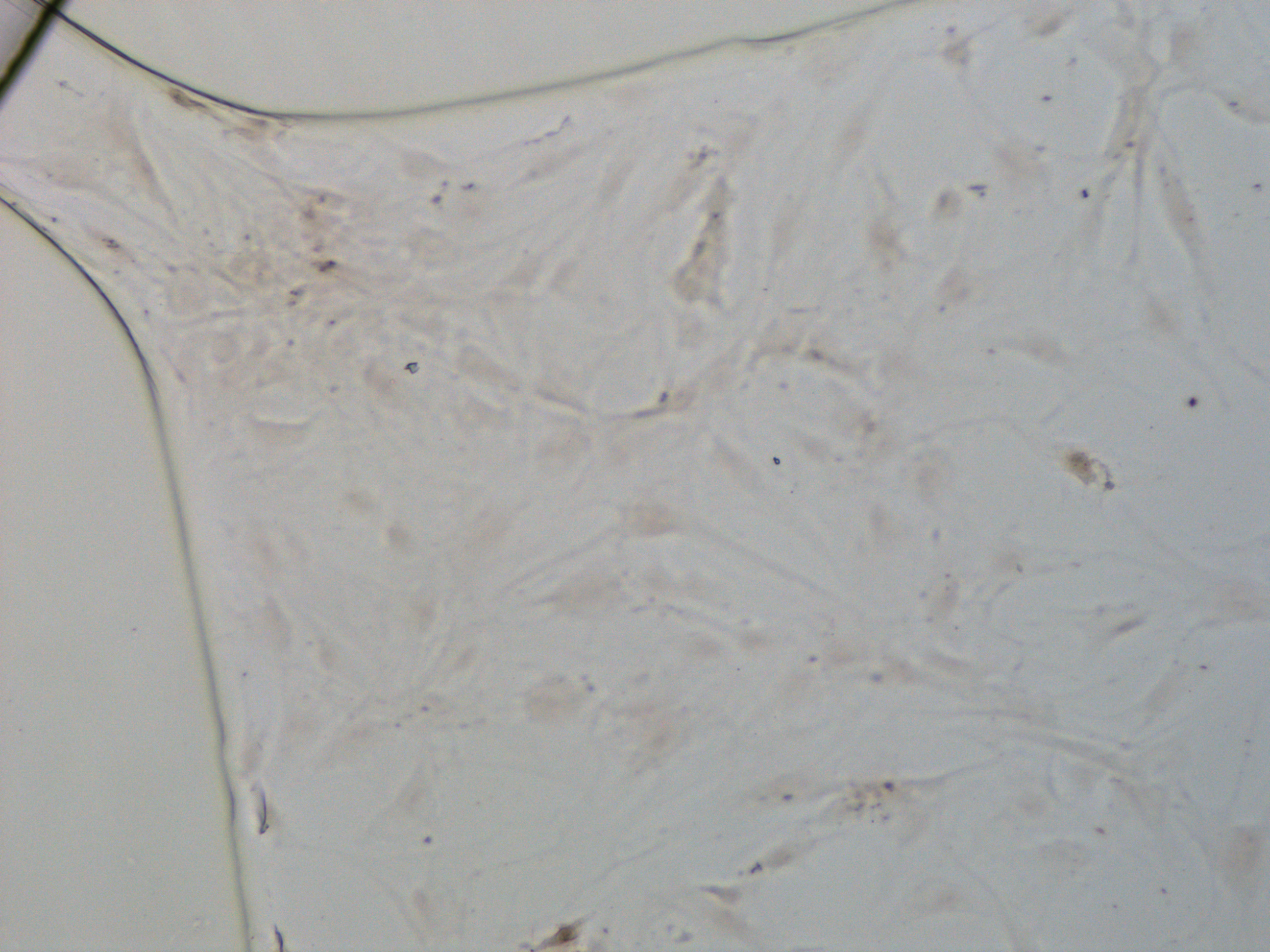

Supplement: S2 File — (ZIP) [file pone.0334482.s002.zip › Alrizarin 100-day 7-NS-ASC1.tif]

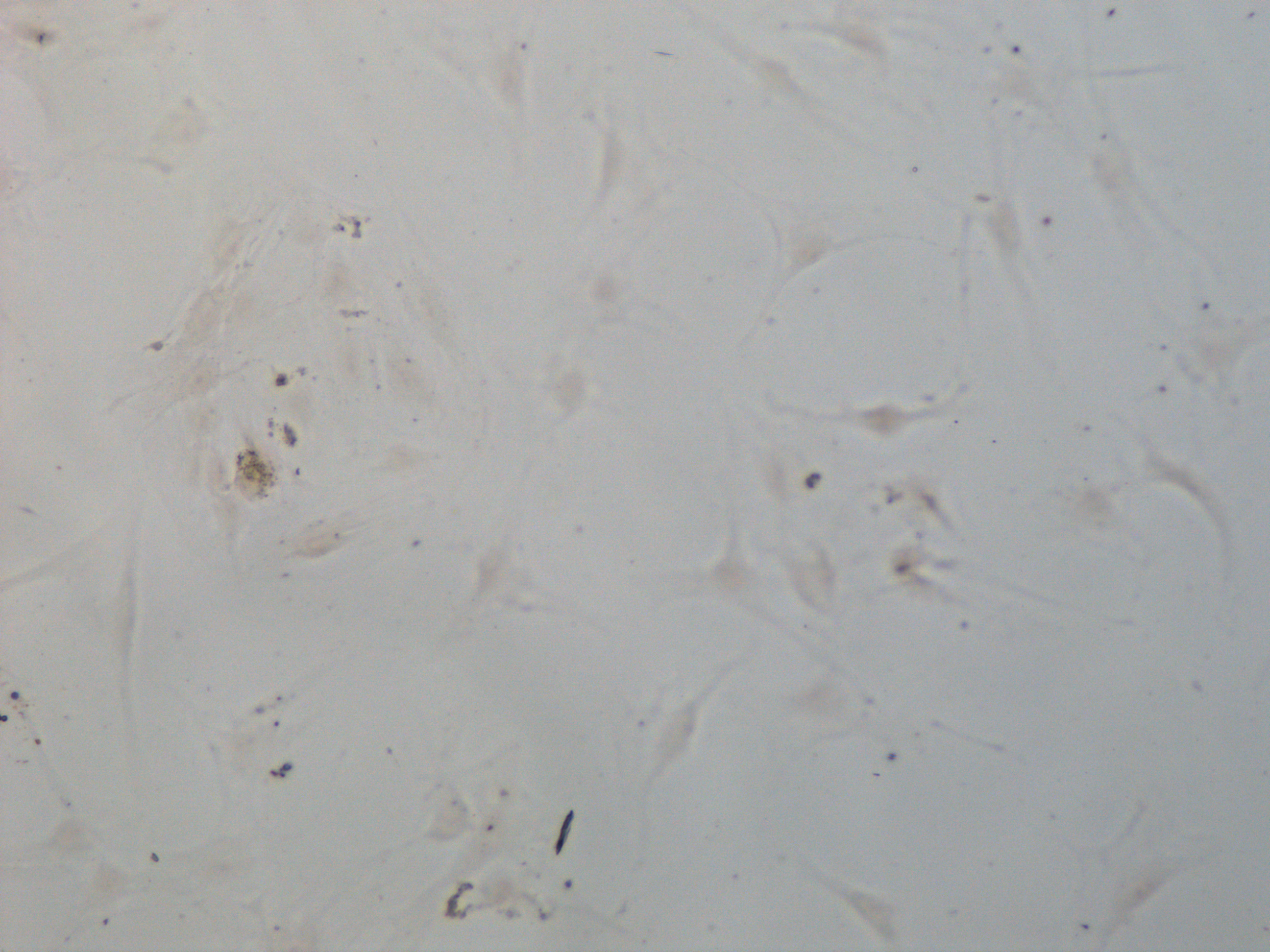

Supplement: S2 File — (ZIP) [file pone.0334482.s002.zip › Alrizarin 100-day 7-NS-ASC6.tif]

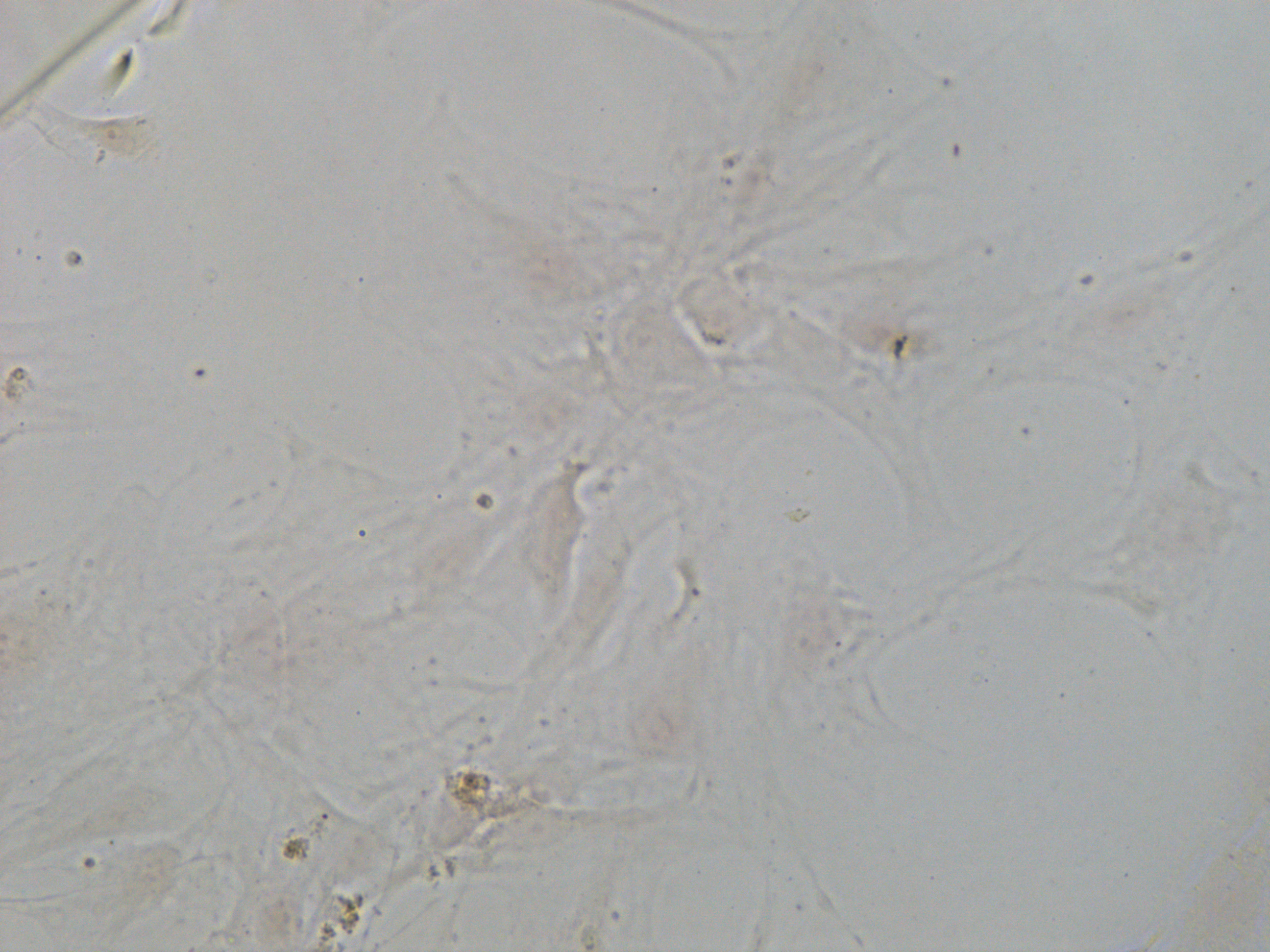

Supplement: S2 File — (ZIP) [file pone.0334482.s002.zip › Alrizarin 200-day 7-NS-ASC8.tif]

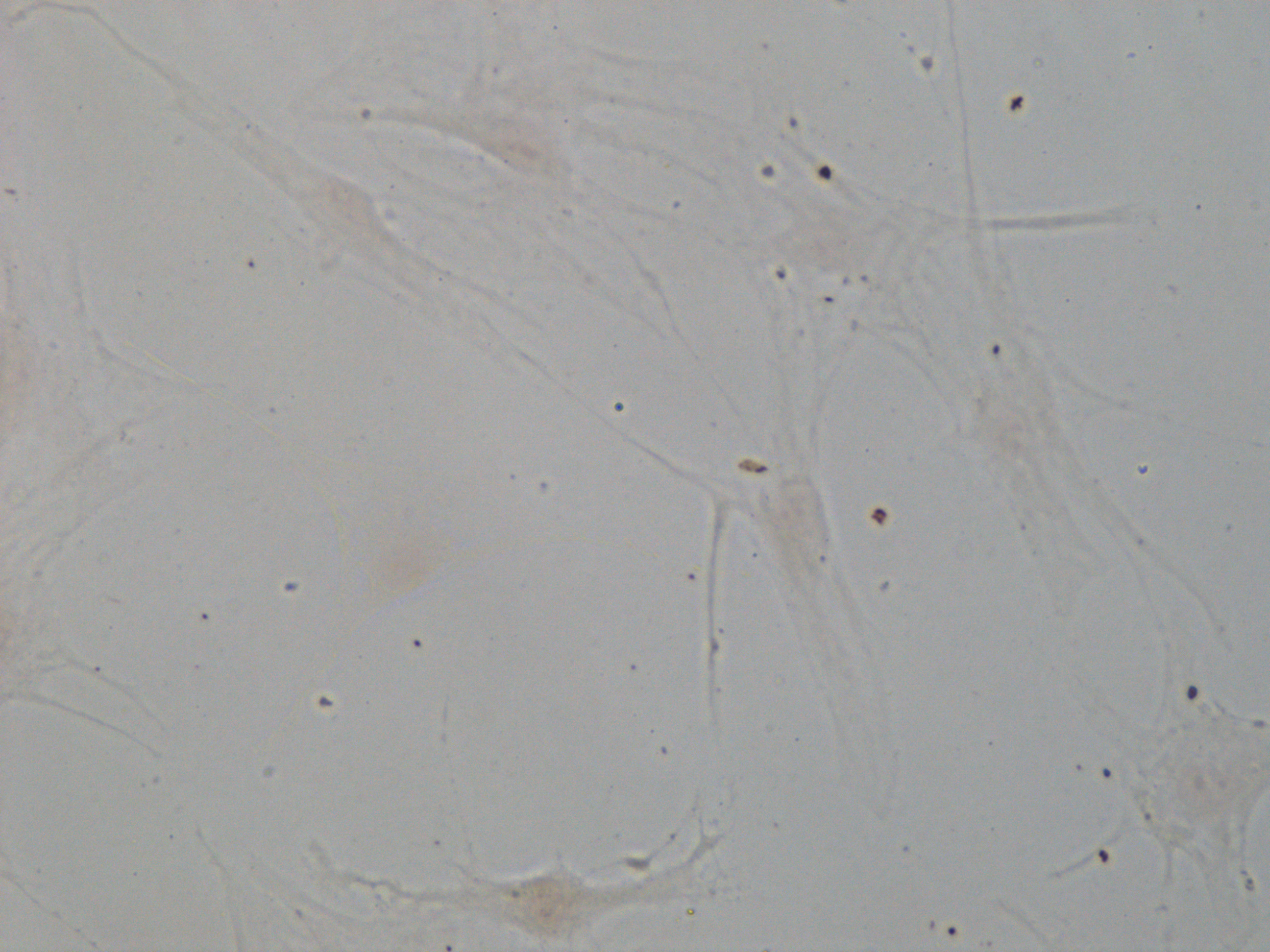

Supplement: S2 File — (ZIP) [file pone.0334482.s002.zip › Alrizarin 200-day 7-NS-ASC9.tif]

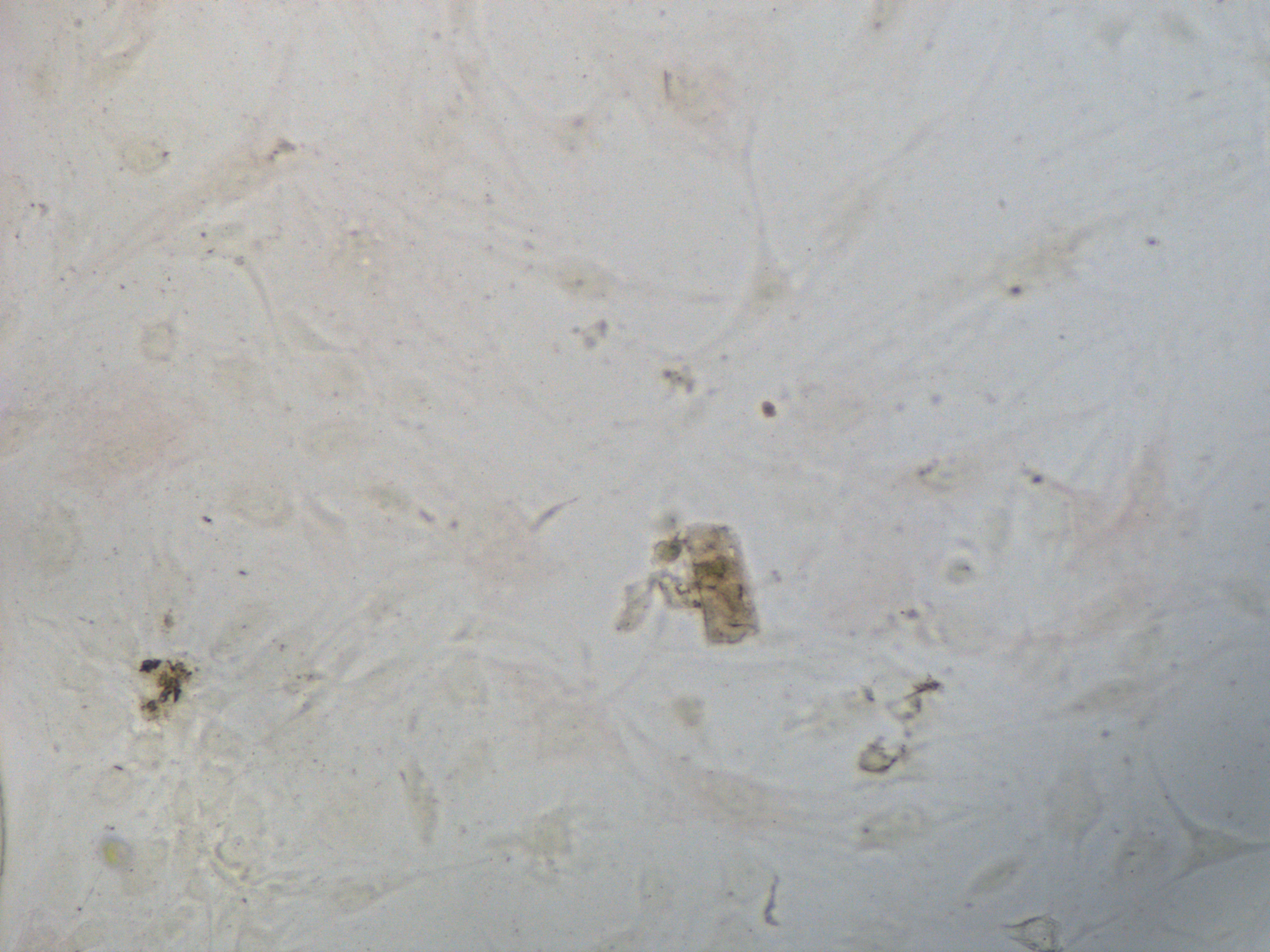

Supplement: S2 File — (ZIP) [file pone.0334482.s002.zip › AR100-day 7-NS-ASC6.tif]

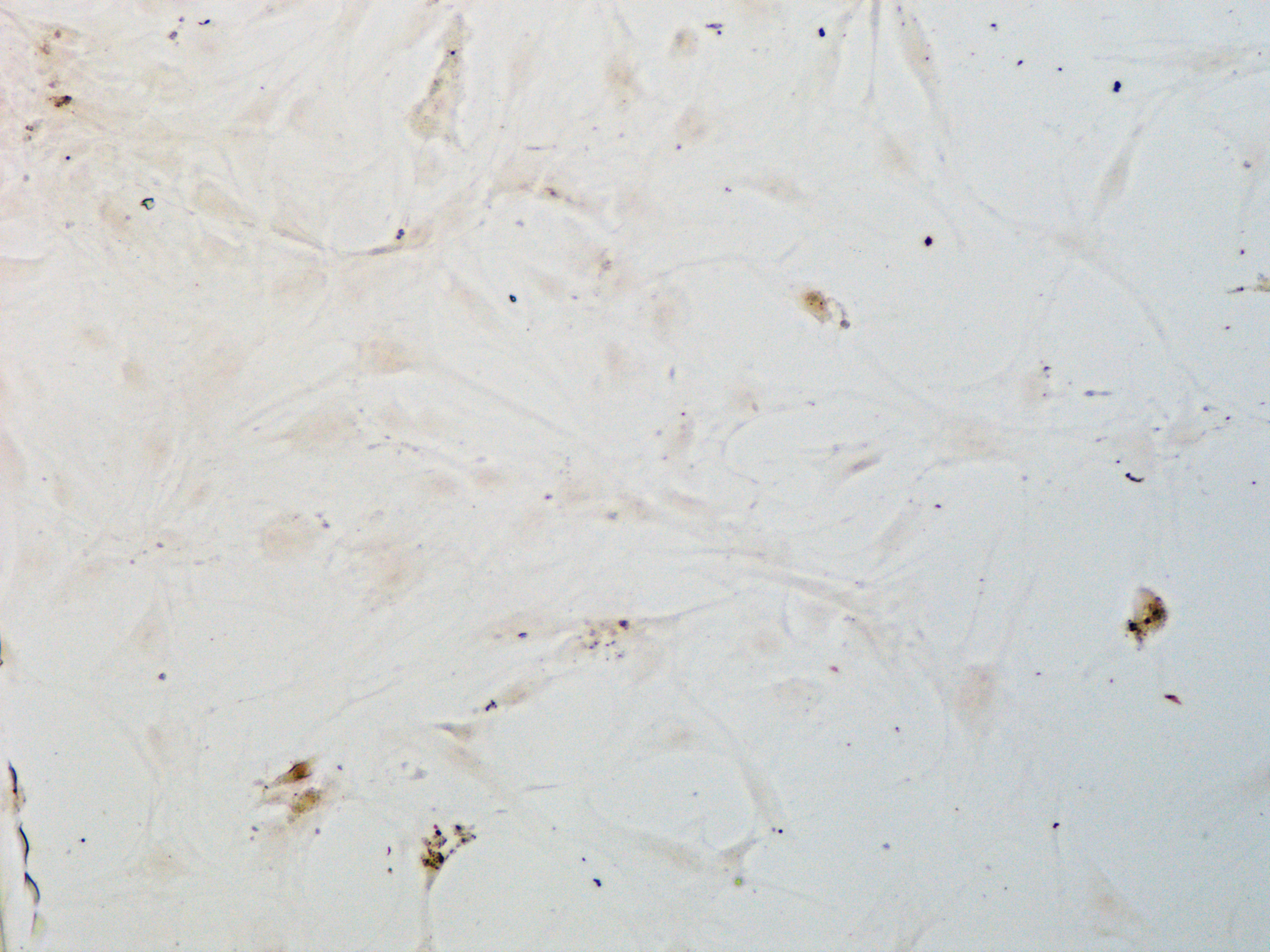

Supplement: S3 File — (ZIP) [file pone.0334482.s003.zip › Sti/11-Alrizarin 100-day 7-Sti-ASC2.tif]

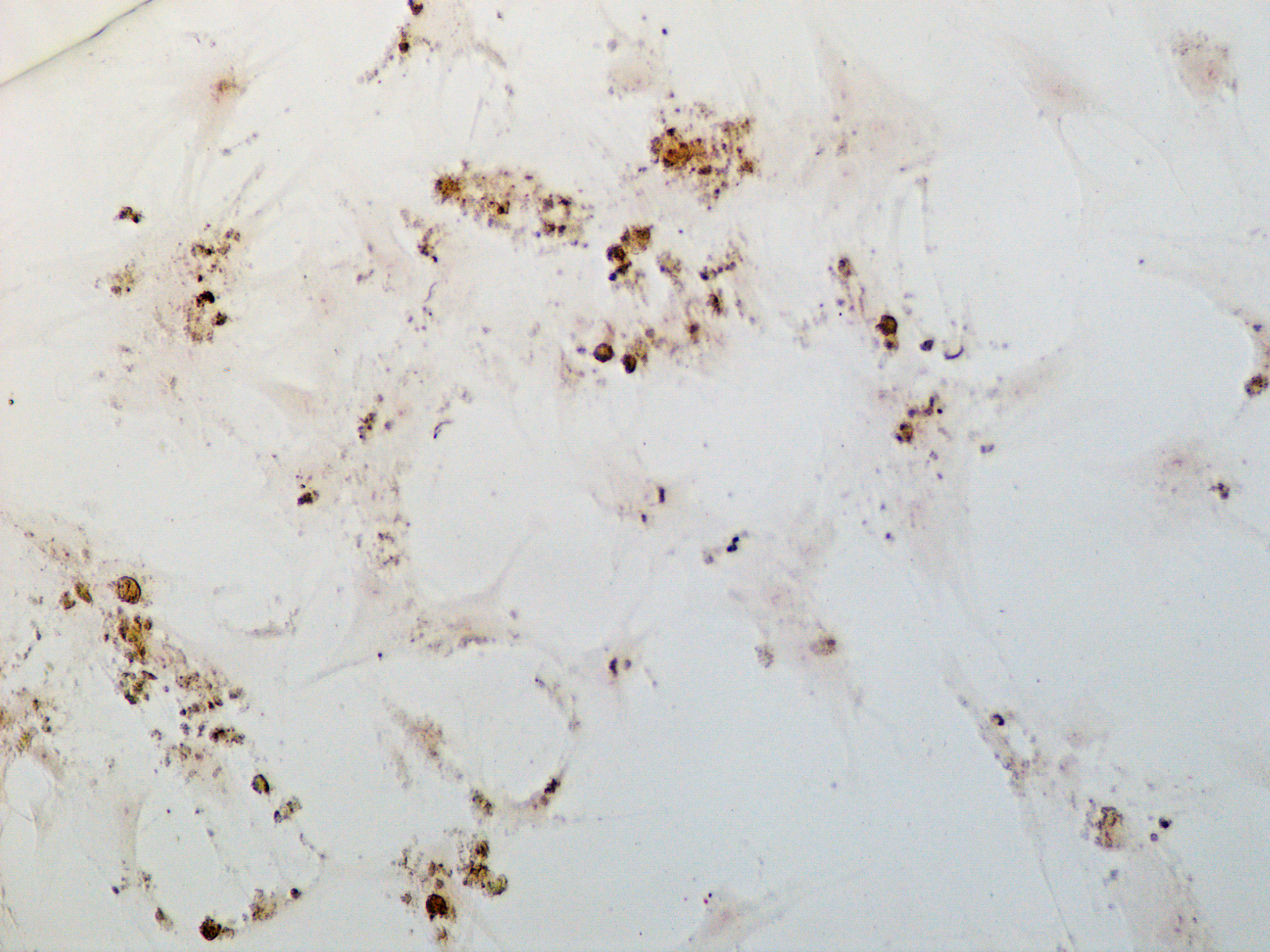

Supplement: S3 File — (ZIP) [file pone.0334482.s003.zip › Sti/11-Alrizarin-day 7-Sti-MSC2.tif]

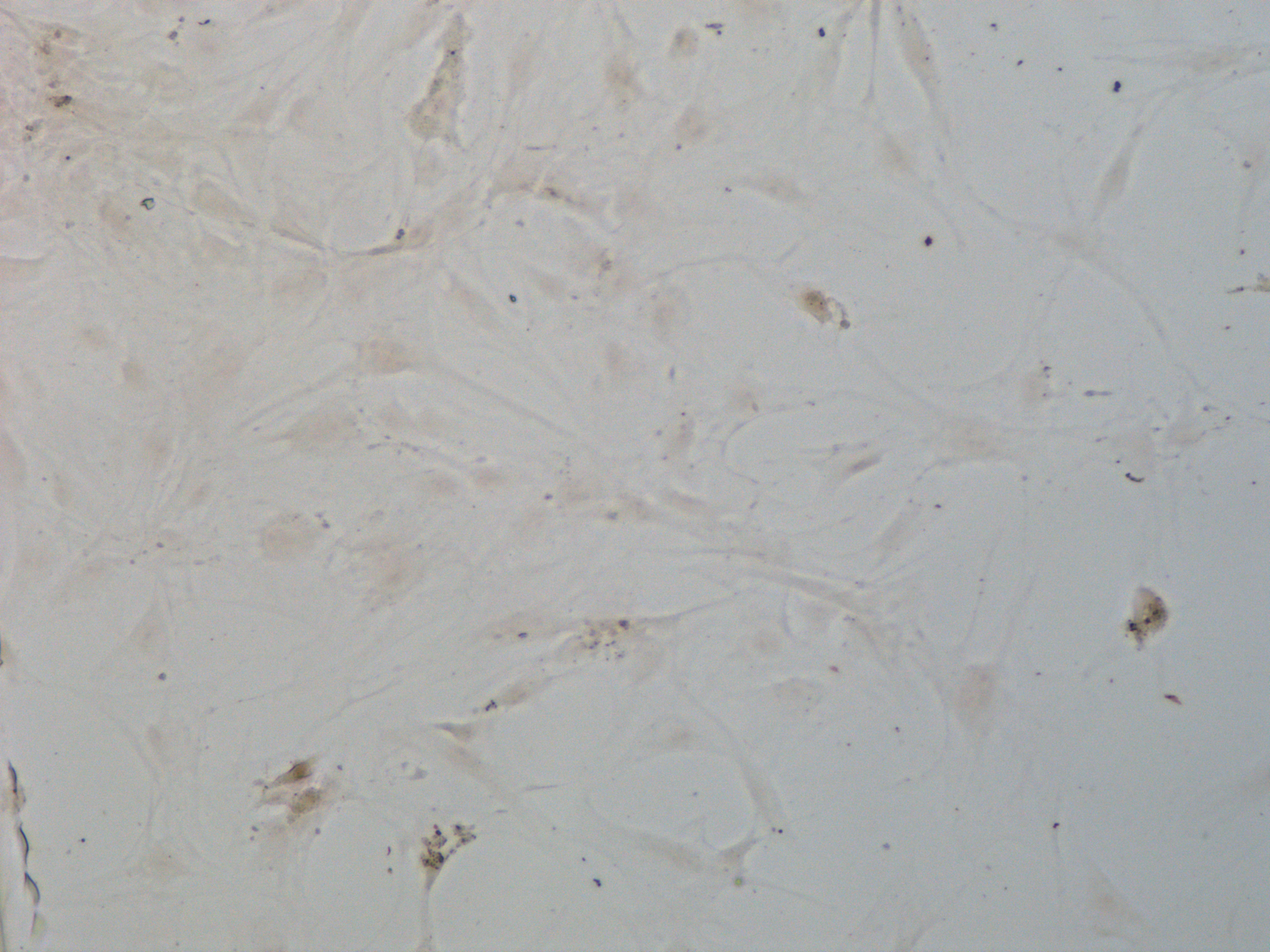

Supplement: S3 File — (ZIP) [file pone.0334482.s003.zip › Sti/Alrizarin 100-day 7-STi-ASC2.tif]

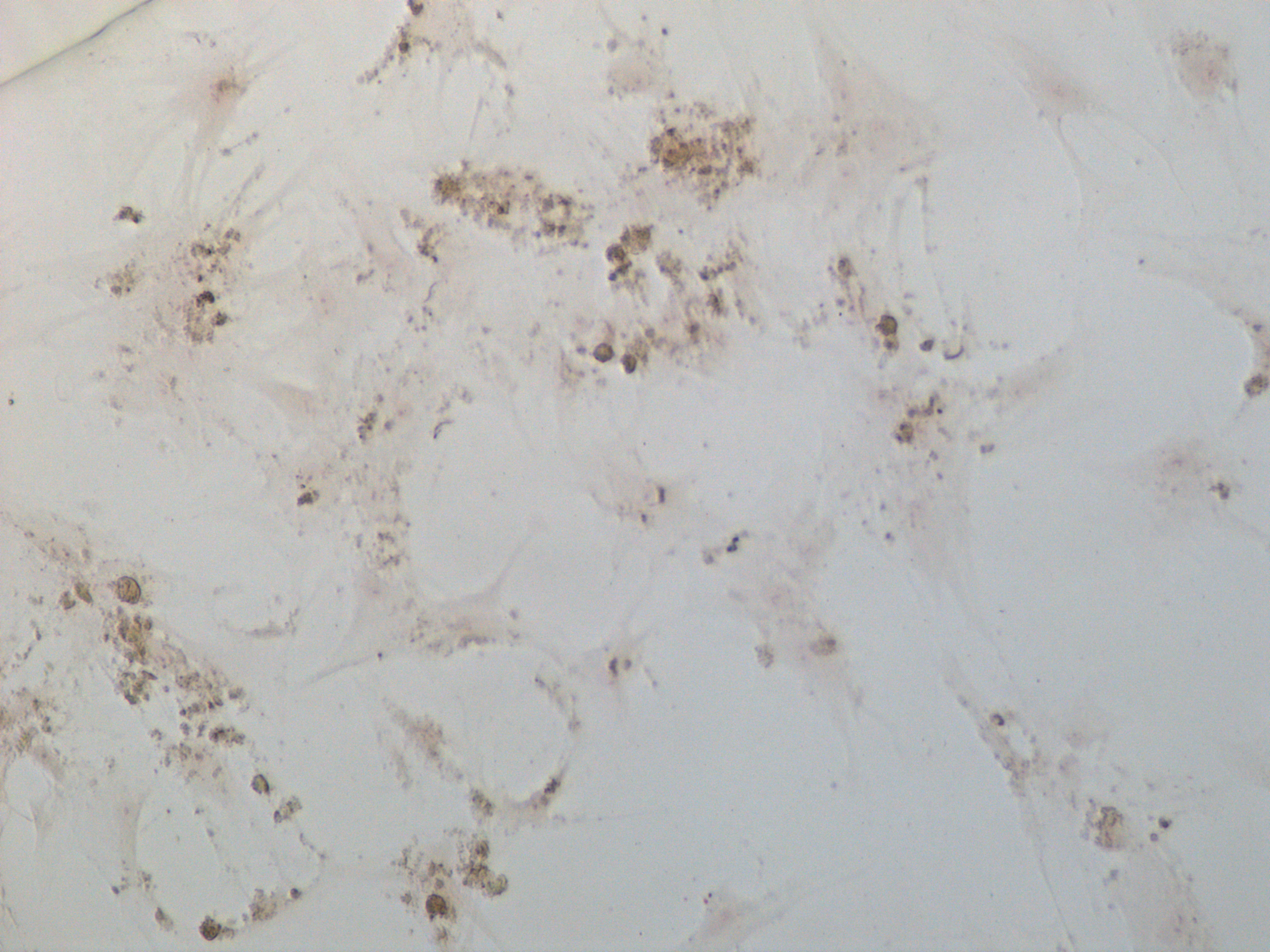

Supplement: S3 File — (ZIP) [file pone.0334482.s003.zip › Sti/Alrizarin-day 7-Sti-MSC2.tif]

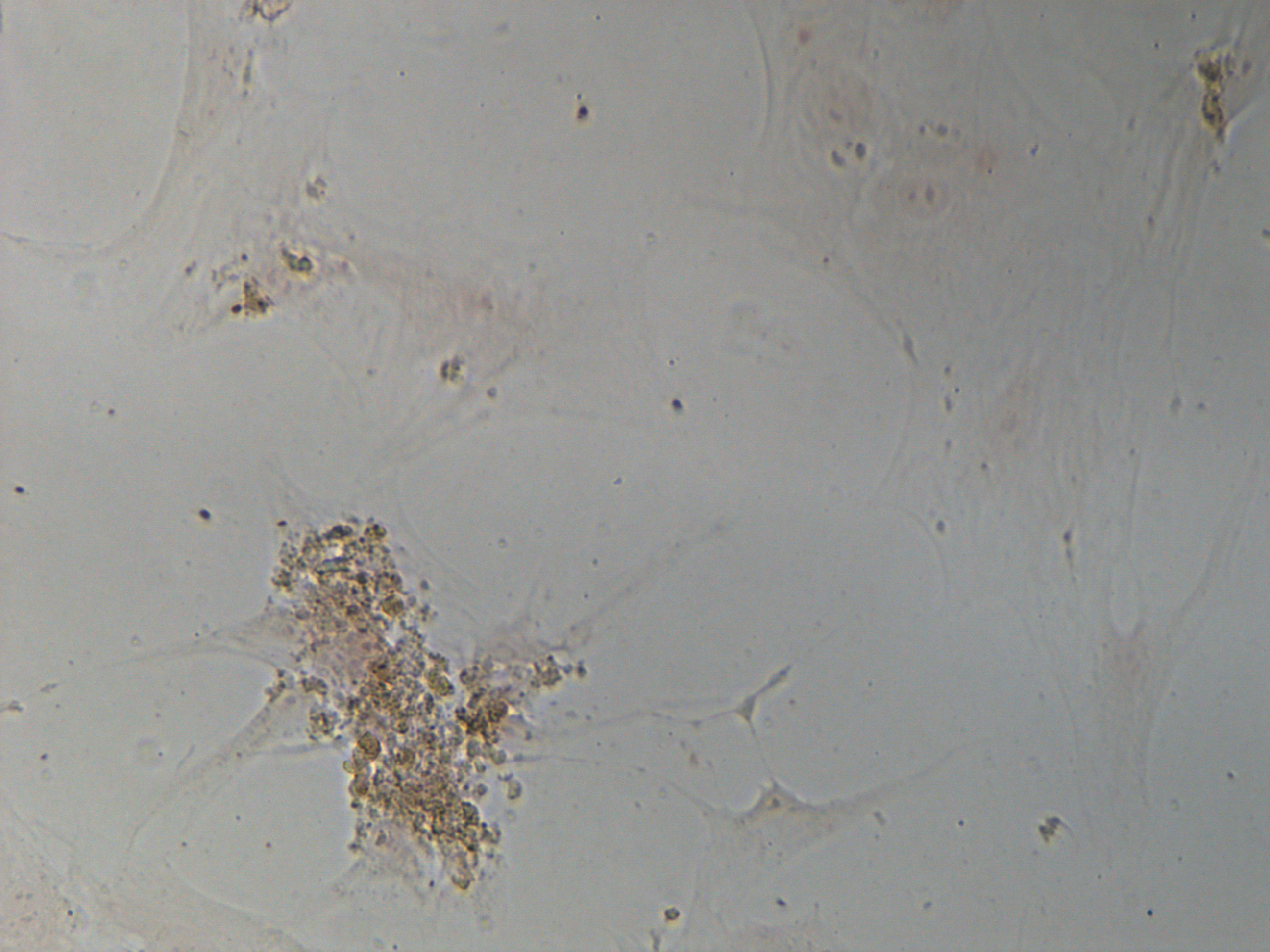

Supplement: S3 File — (ZIP) [file pone.0334482.s003.zip › Sti/Alrizarin100-day 7-Sti-ASC2.tif]

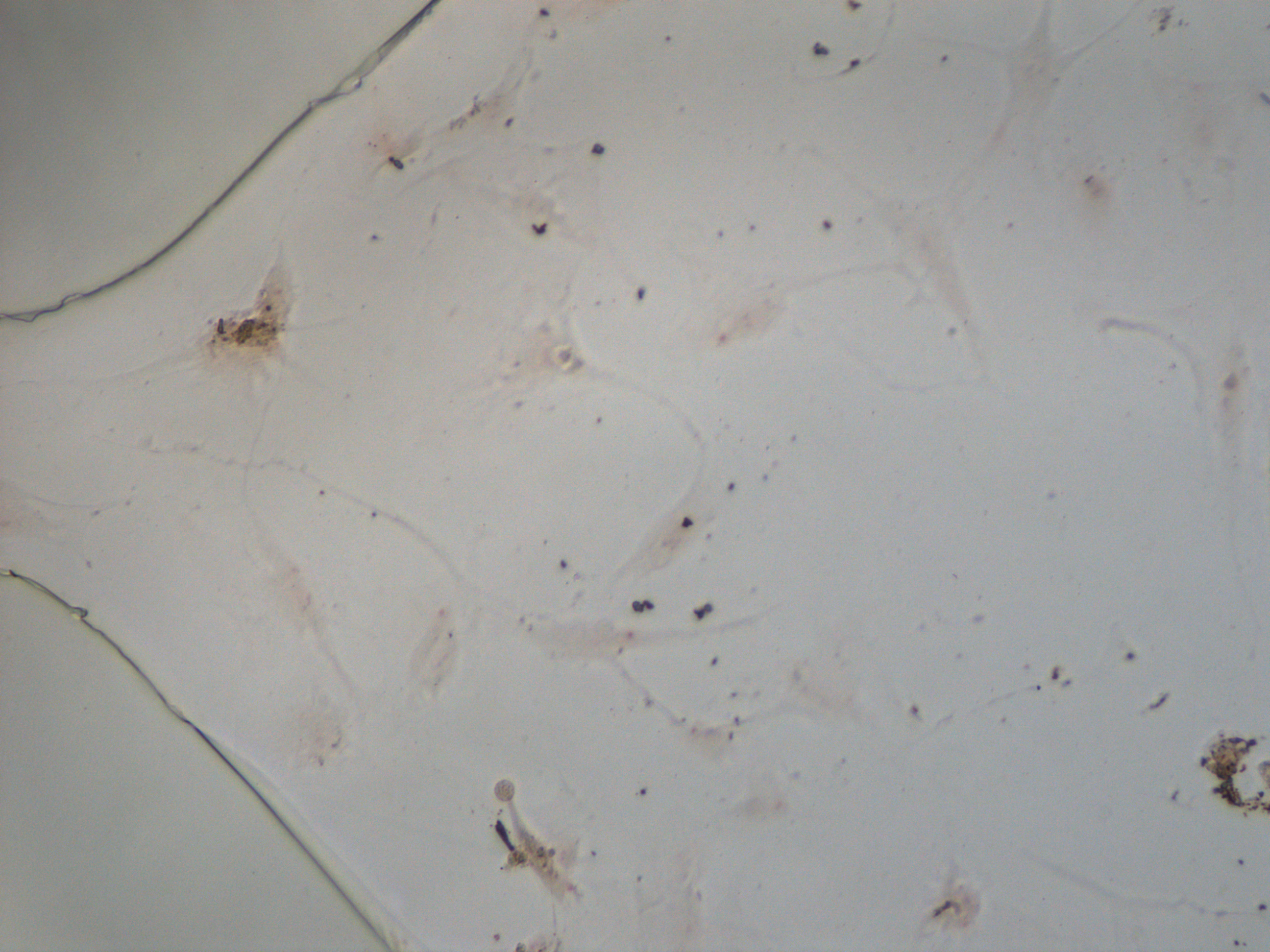

Supplement: S3 File — (ZIP) [file pone.0334482.s003.zip › Sti/Alrizarin100-day 7-Sti-ASC5.tif]

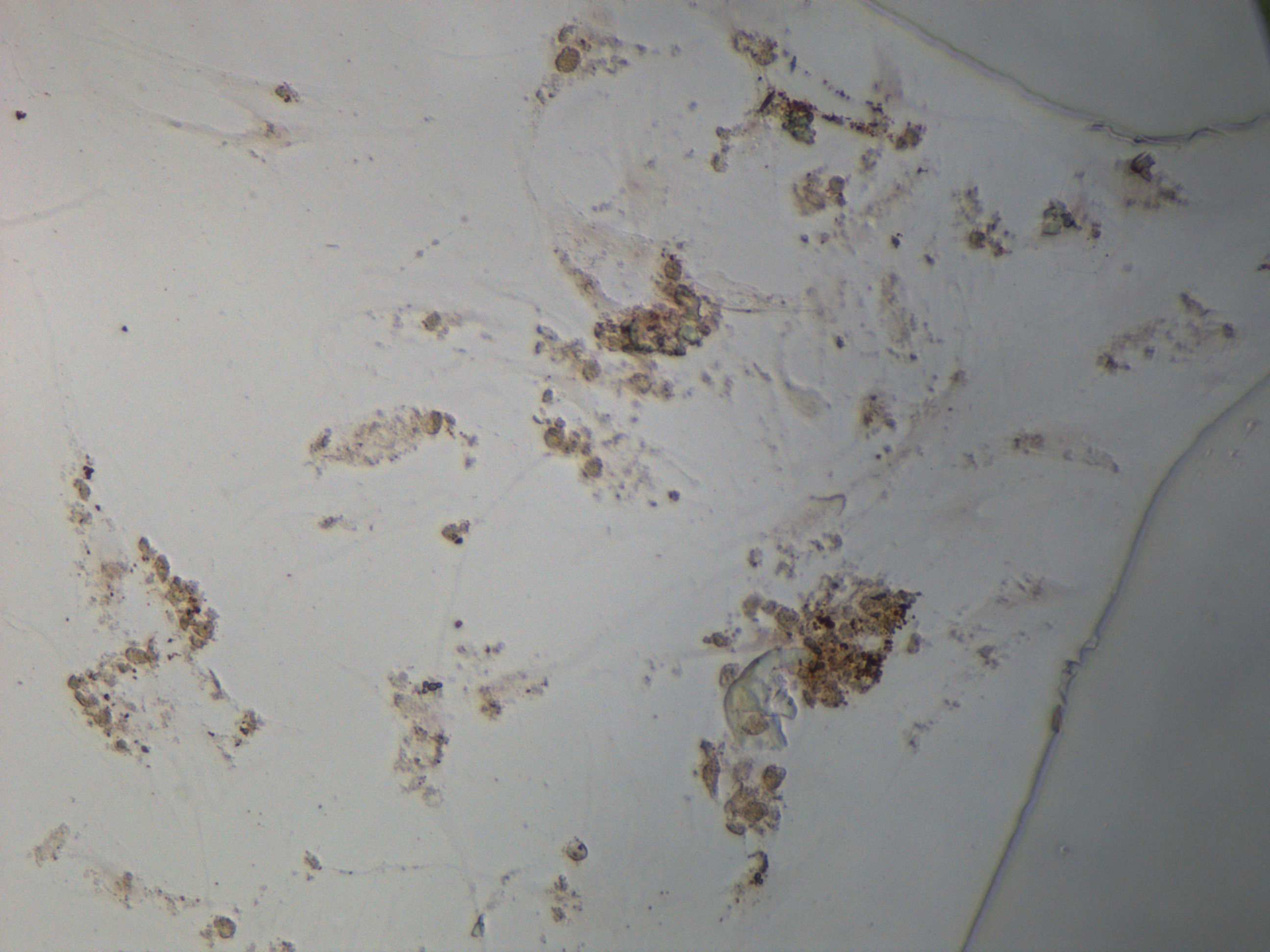

Supplement: S3 File — (ZIP) [file pone.0334482.s003.zip › Sti/Alrizarin100-day 7-Sti-MSC5.tif]

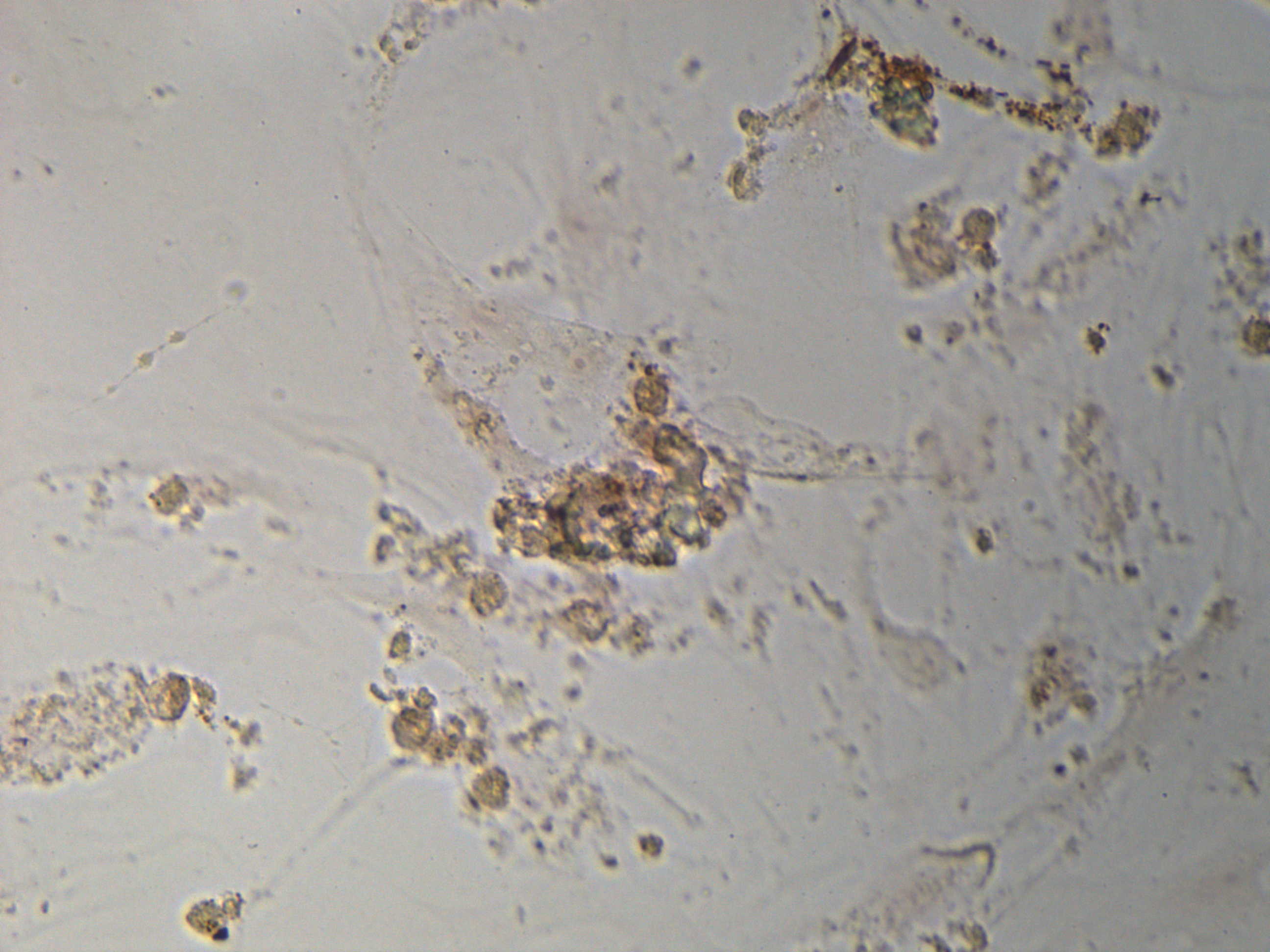

Supplement: S3 File — (ZIP) [file pone.0334482.s003.zip › Sti/Alrizarin200-day 7-Sti-MSC8.tif]

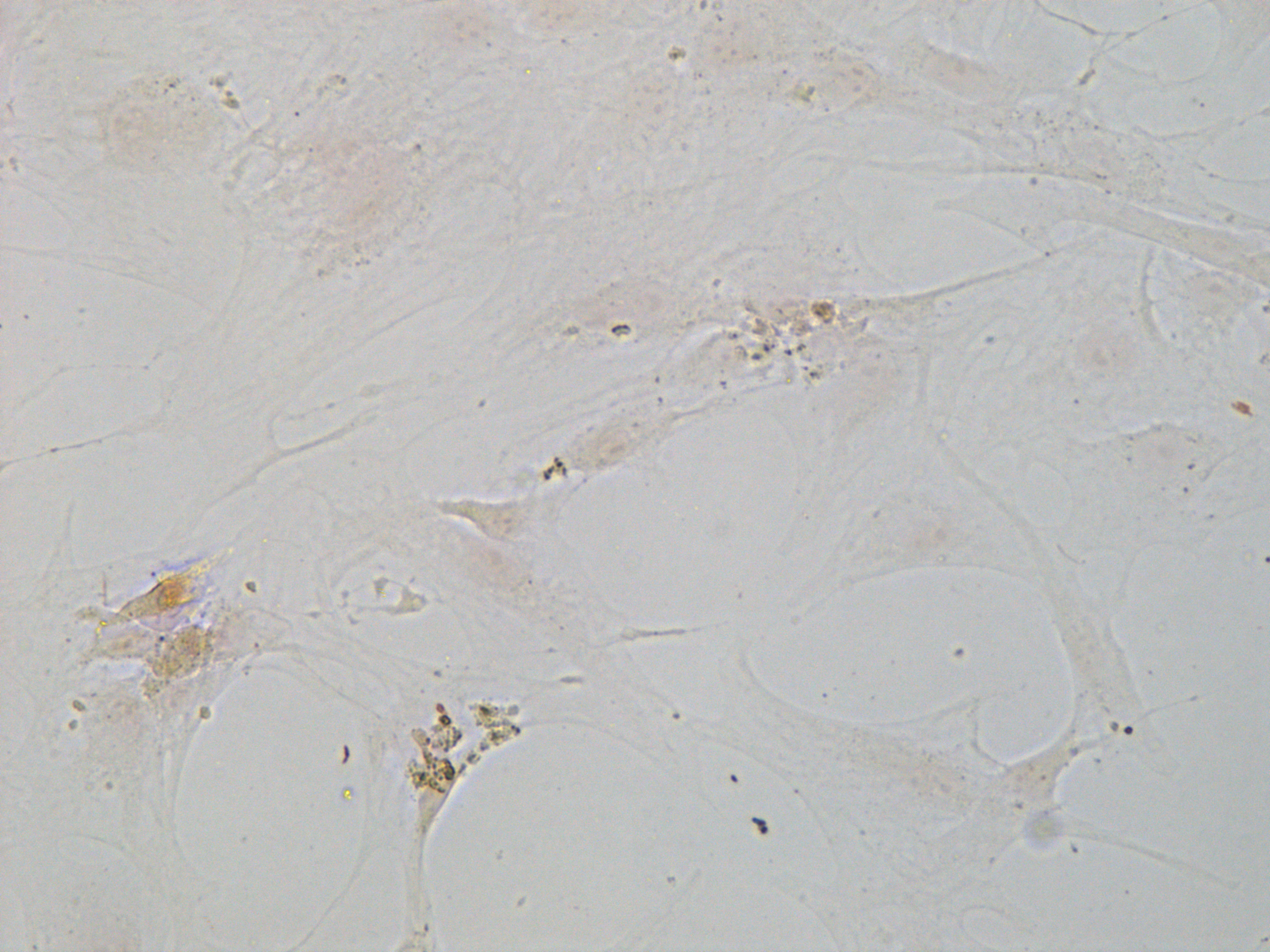

Supplement: S3 File — (ZIP) [file pone.0334482.s003.zip › Sti/AR 200-day 7-S-ASC4.tif]

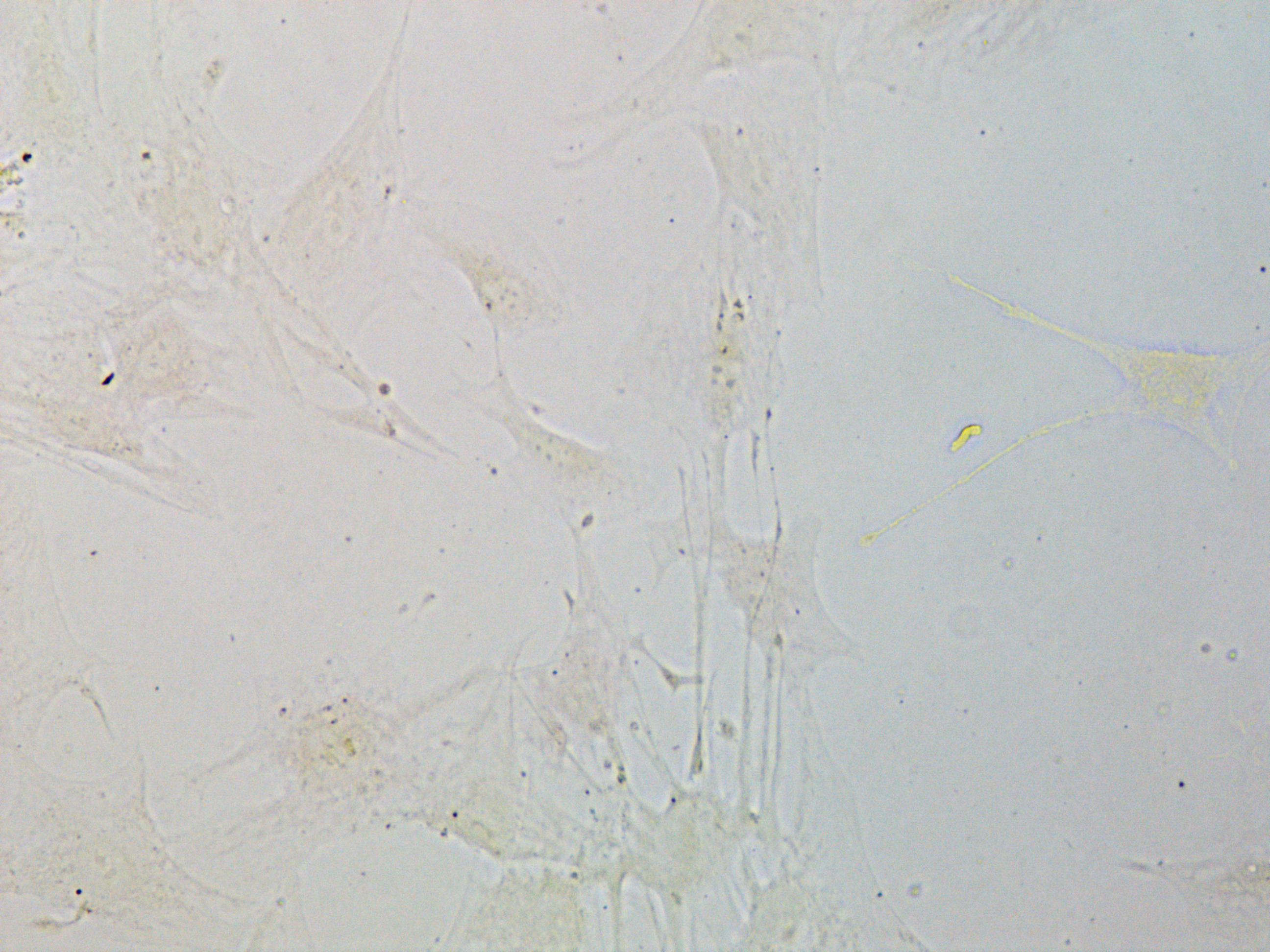

Supplement: S4 File — (ZIP) [file pone.0334482.s004.zip › Non Sti/11-ALP 200-day 7-NS-ASC7.tif]

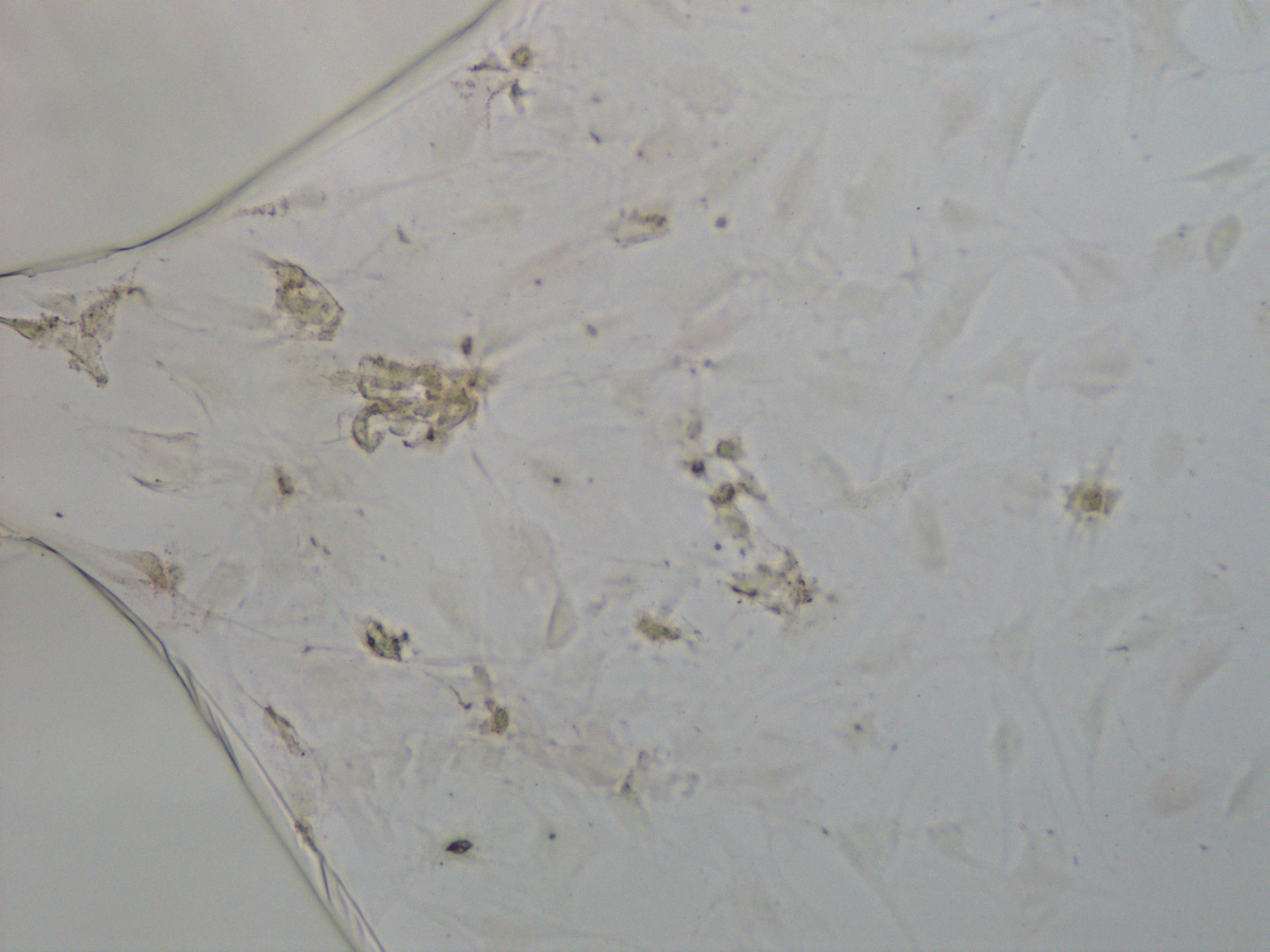

Supplement: S4 File — (ZIP) [file pone.0334482.s004.zip › Non Sti/ALP 100-day 7-NS-ASC1.tif]

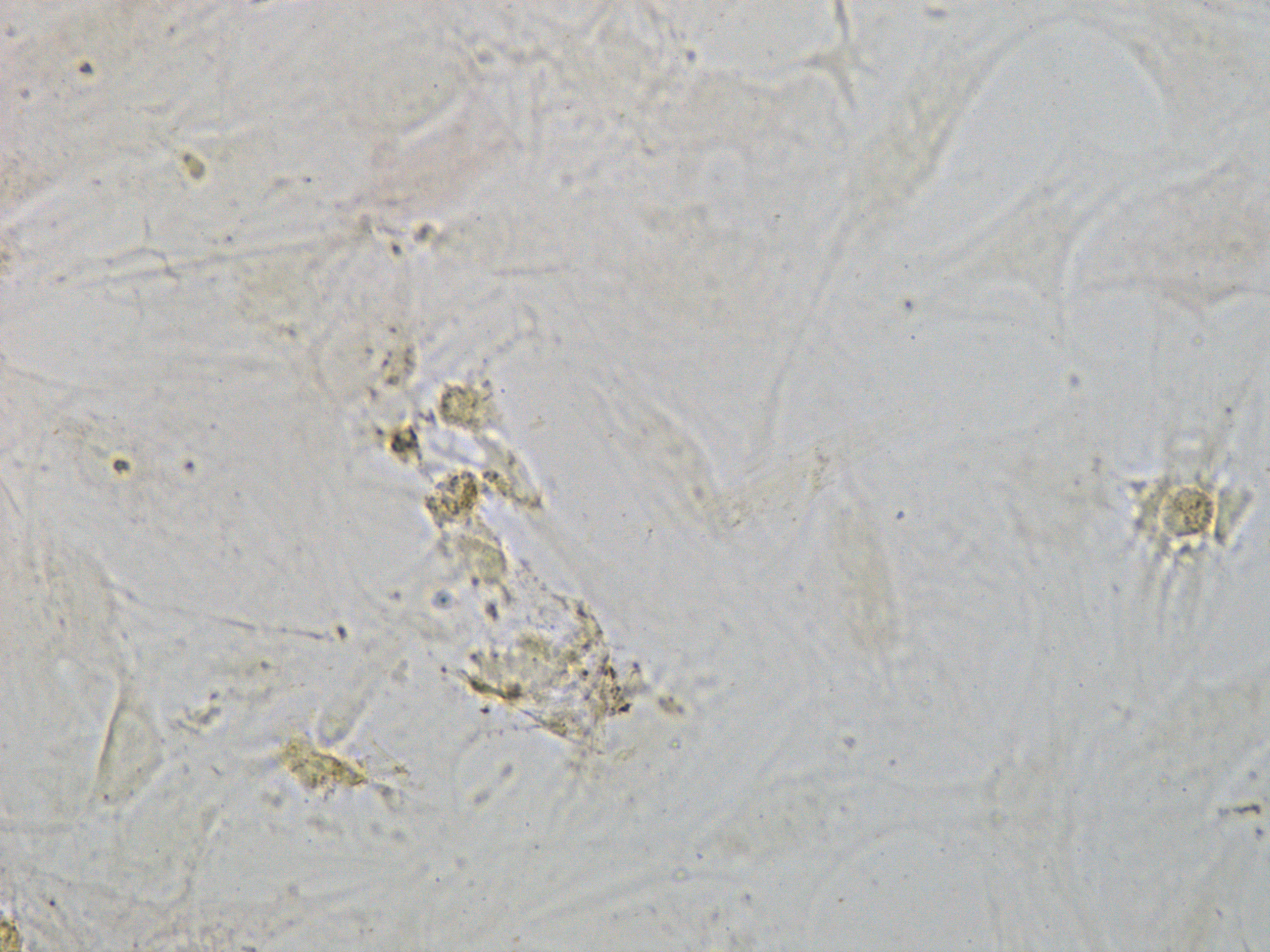

Supplement: S4 File — (ZIP) [file pone.0334482.s004.zip › Non Sti/ALP 200-day 7-NS-ASC4.tif]

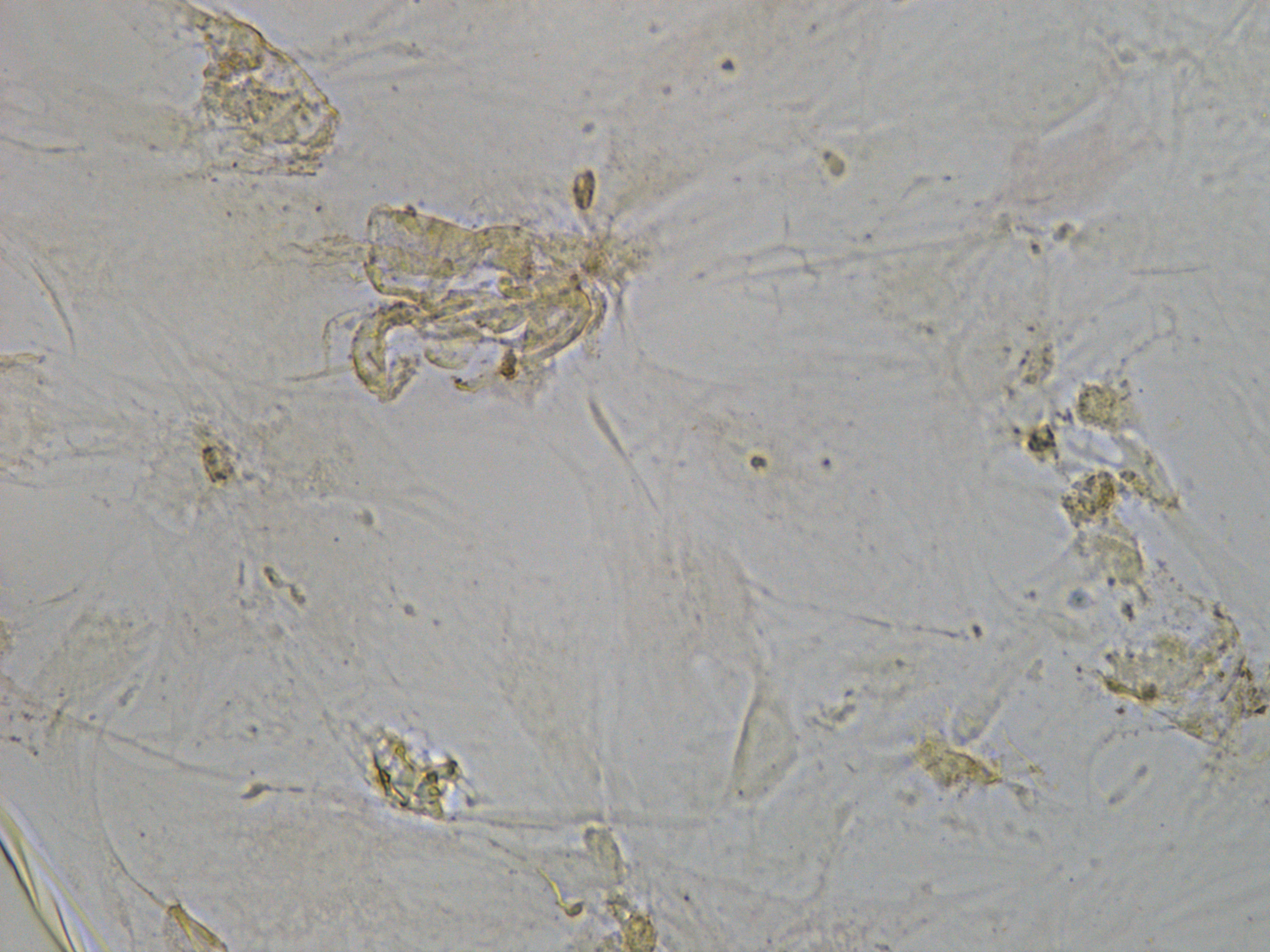

Supplement: S4 File — (ZIP) [file pone.0334482.s004.zip › Non Sti/ALP 200-day 7-NS-ASC5.tif]

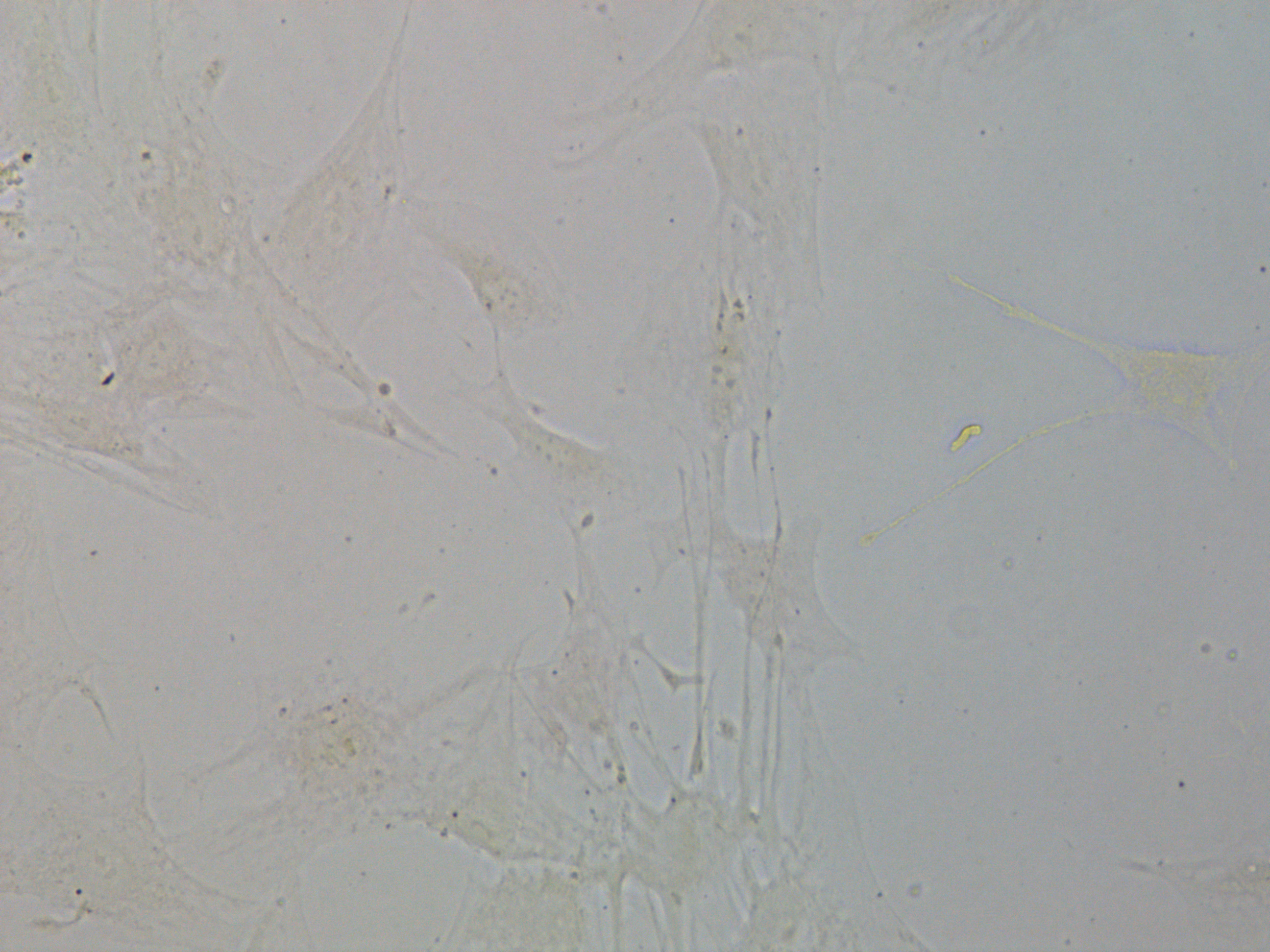

Supplement: S4 File — (ZIP) [file pone.0334482.s004.zip › Non Sti/ALP 200-day 7-NS-ASC7.tif]

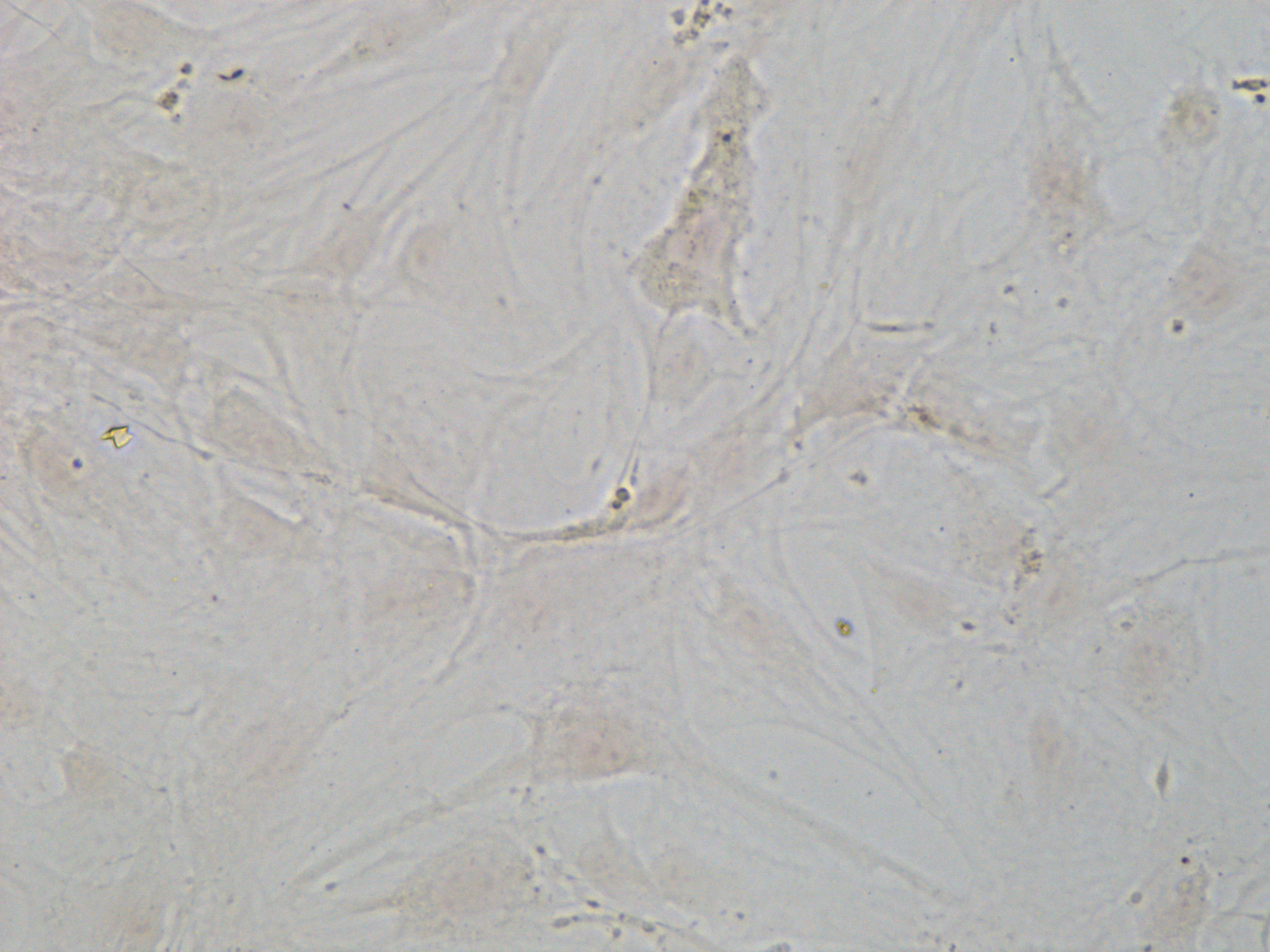

Supplement: S4 File — (ZIP) [file pone.0334482.s004.zip › Non Sti/ALP 200-day 7-NS-MSC3.tif]

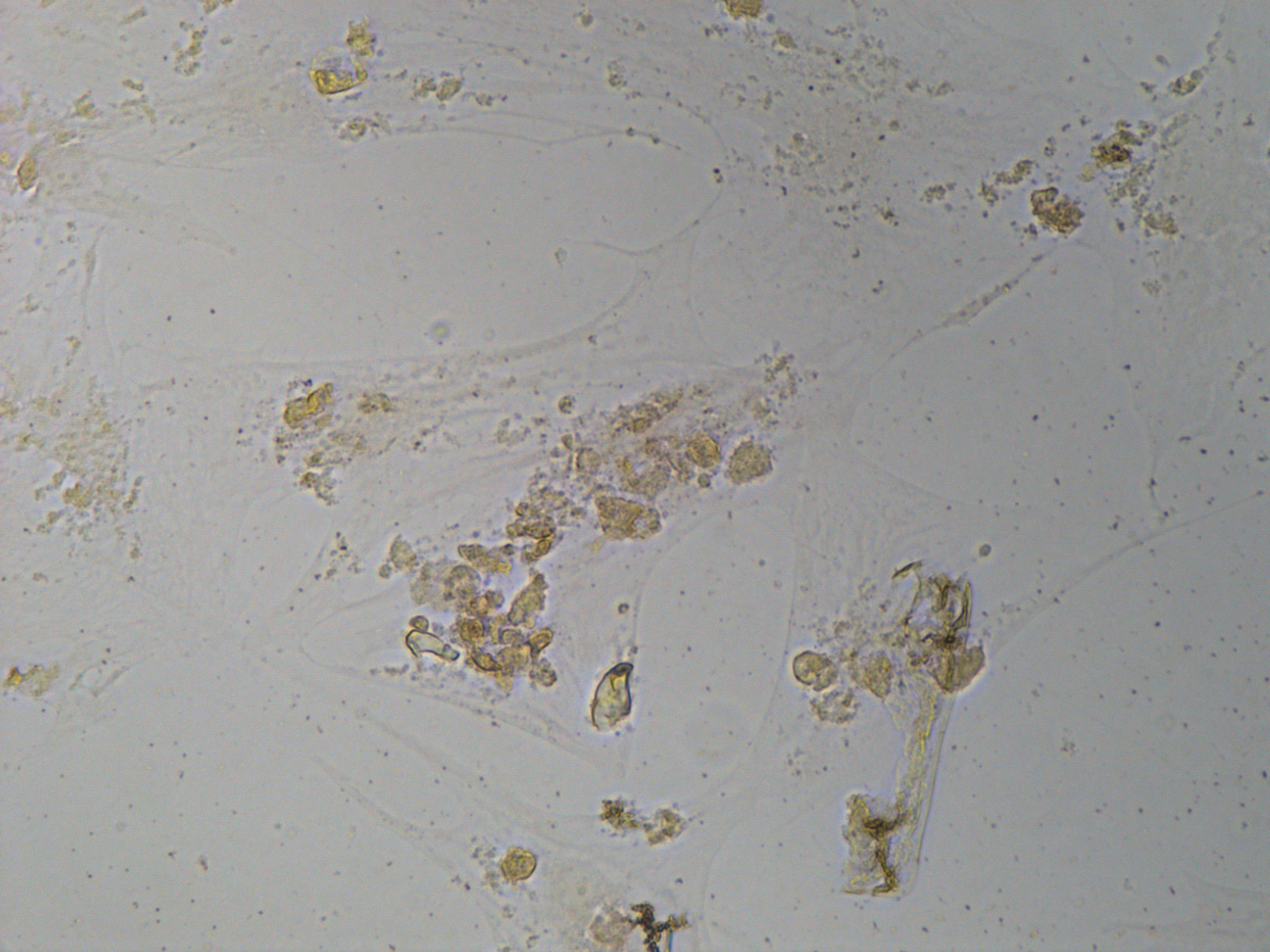

Supplement: S4 File — (ZIP) [file pone.0334482.s004.zip › Non Sti/ALP200-day 7-NS-MSC12.tif]

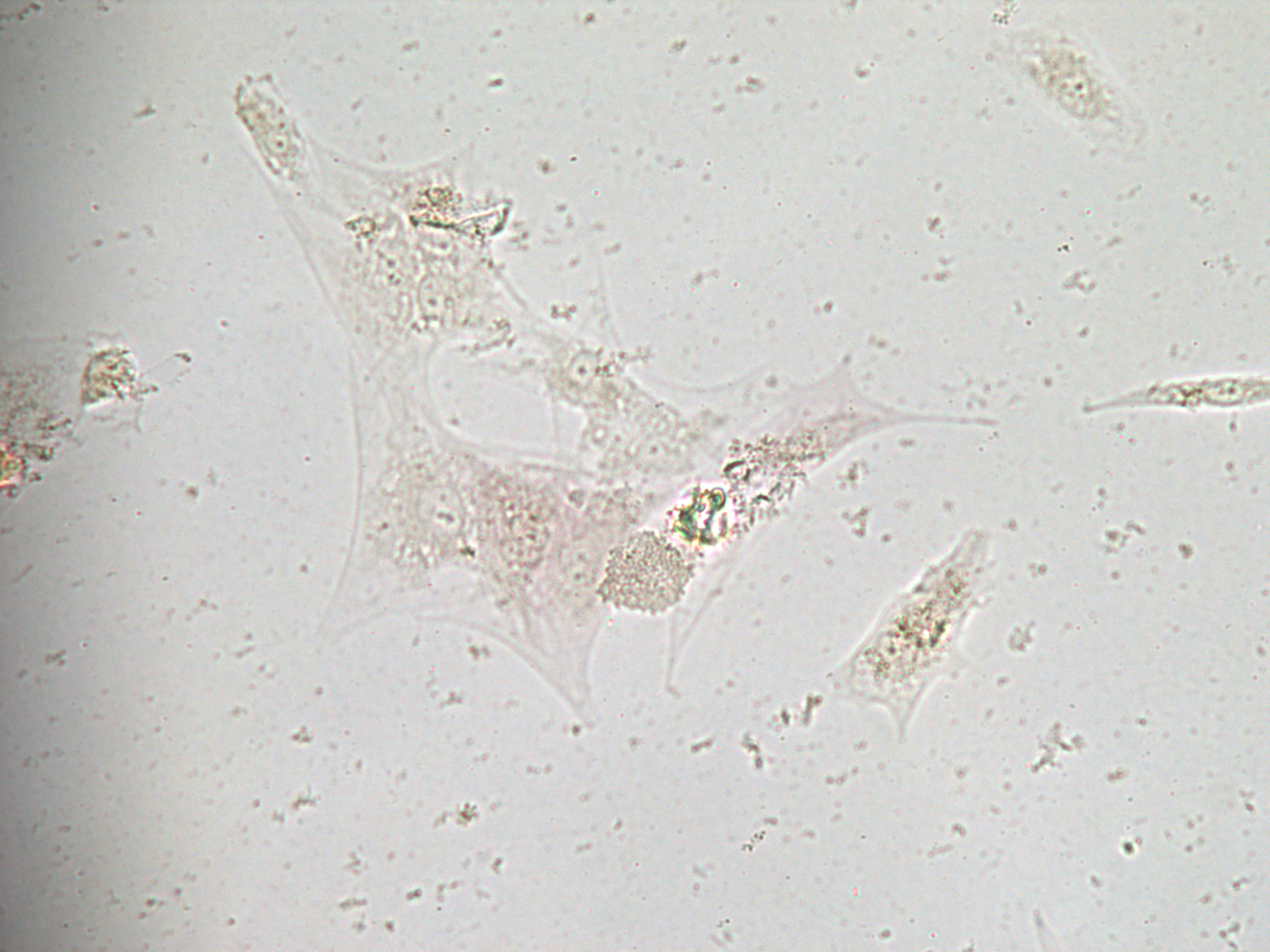

Supplement: S4 File — (ZIP) [file pone.0334482.s004.zip › Non Sti/contorl ASC-ALP 11.tif]

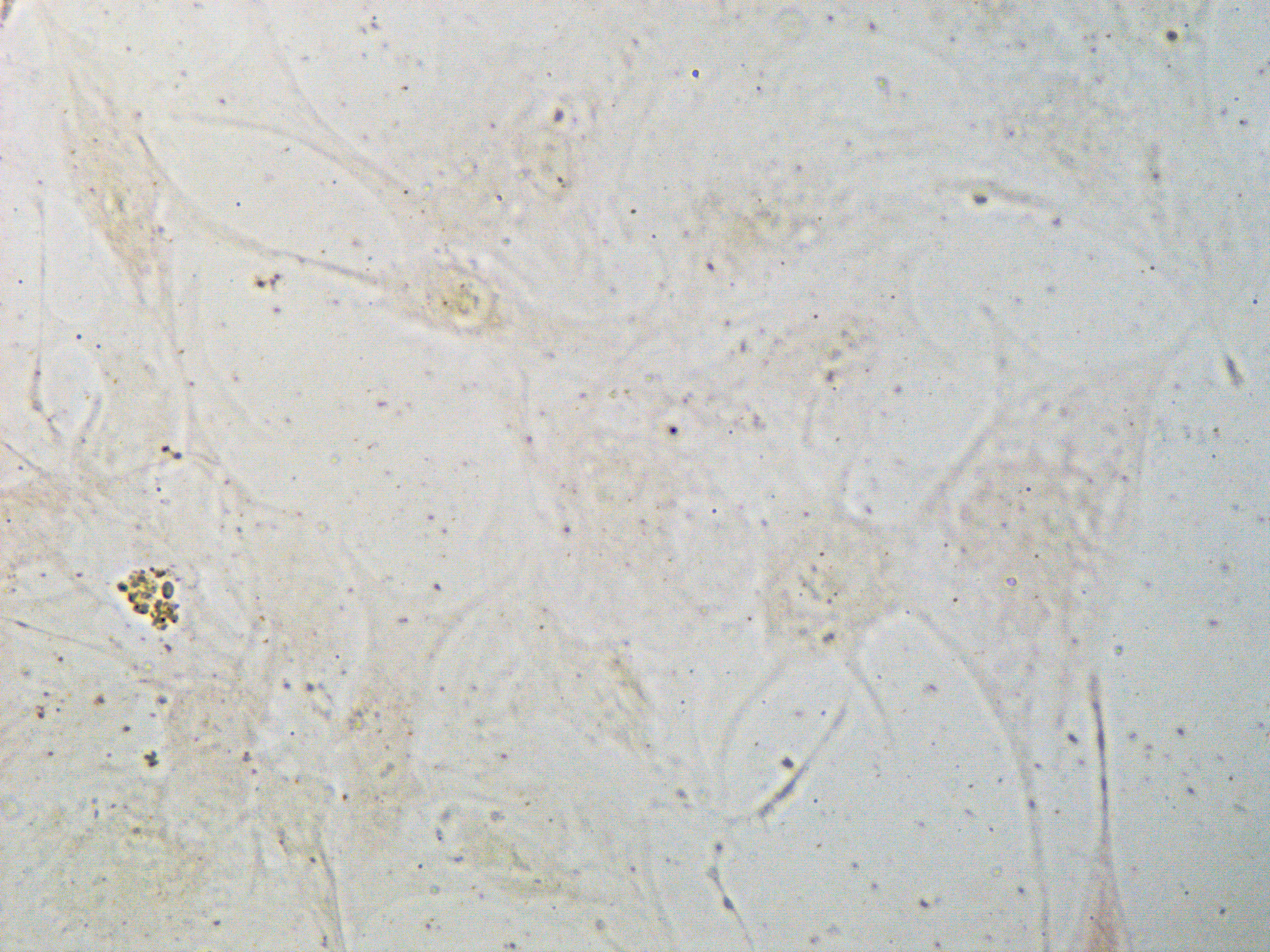

Supplement: S5 File — (ZIP) [file pone.0334482.s005.zip › Sti/11-ALP200-day 7-Sti-ASC4.tif]

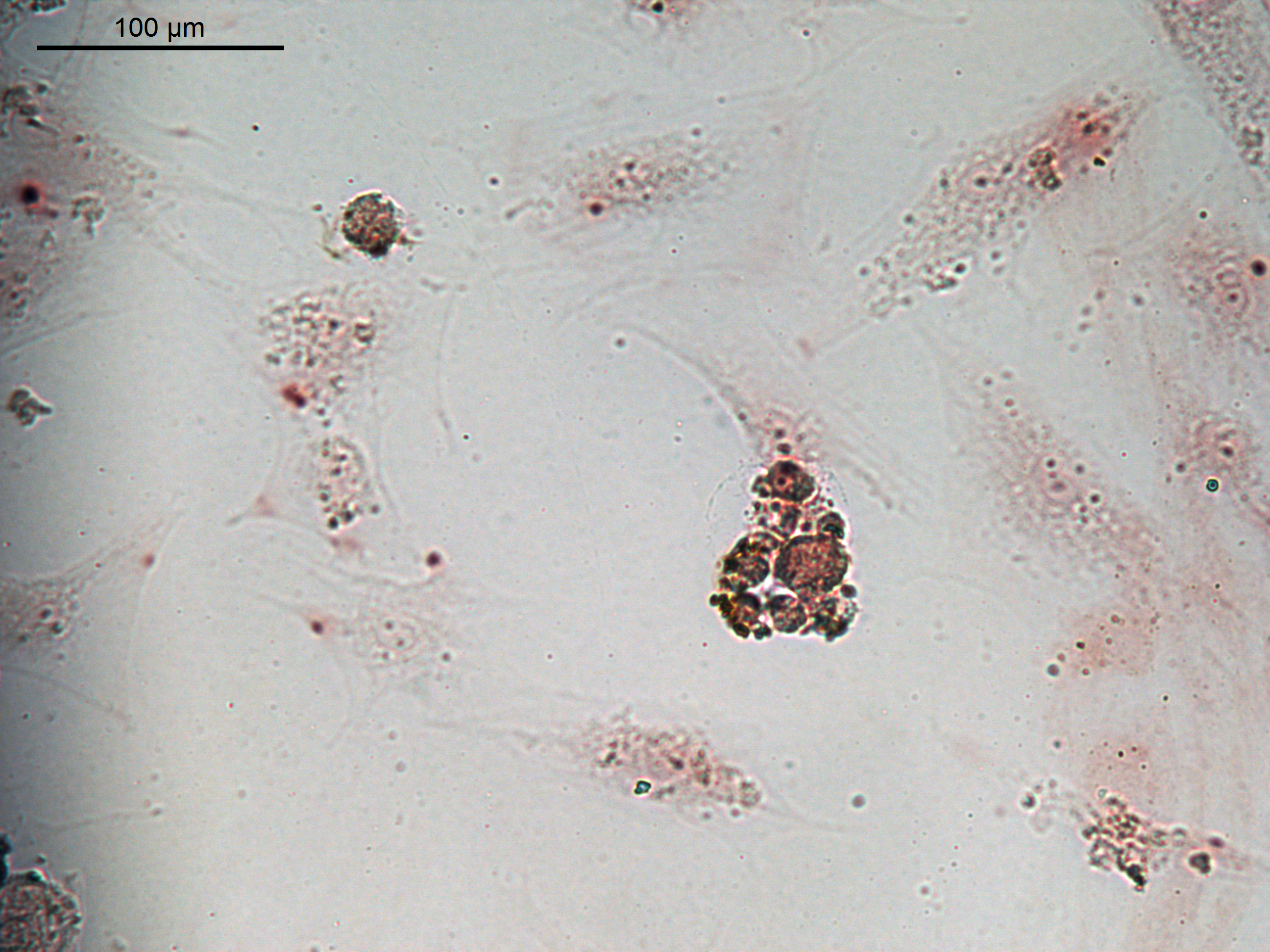

Supplement: S5 File — (ZIP) [file pone.0334482.s005.zip › Sti/11-Sti- MSC ALP.tif]

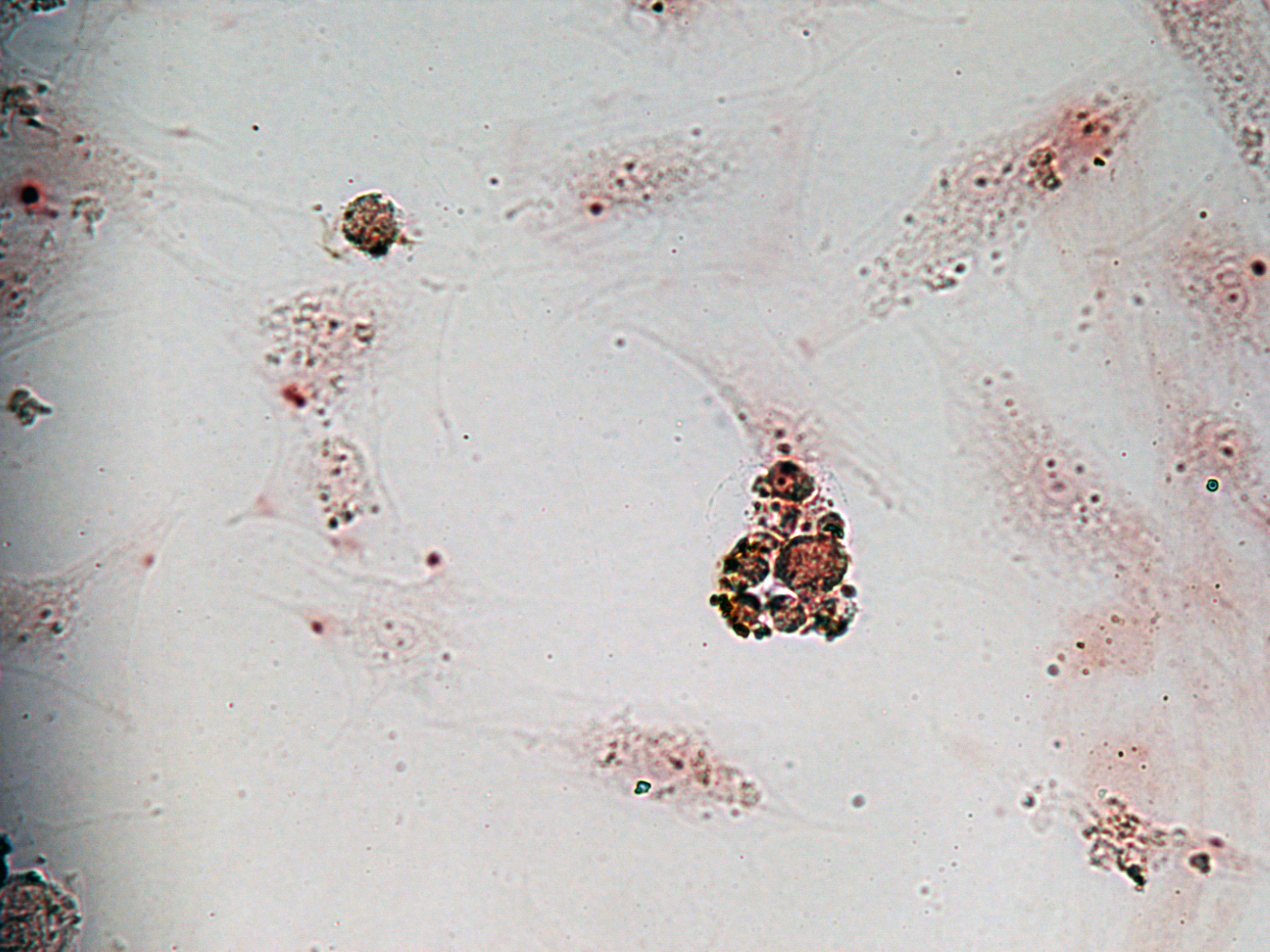

Supplement: S5 File — (ZIP) [file pone.0334482.s005.zip › Sti/12-Sti- MSC ALP.tif]

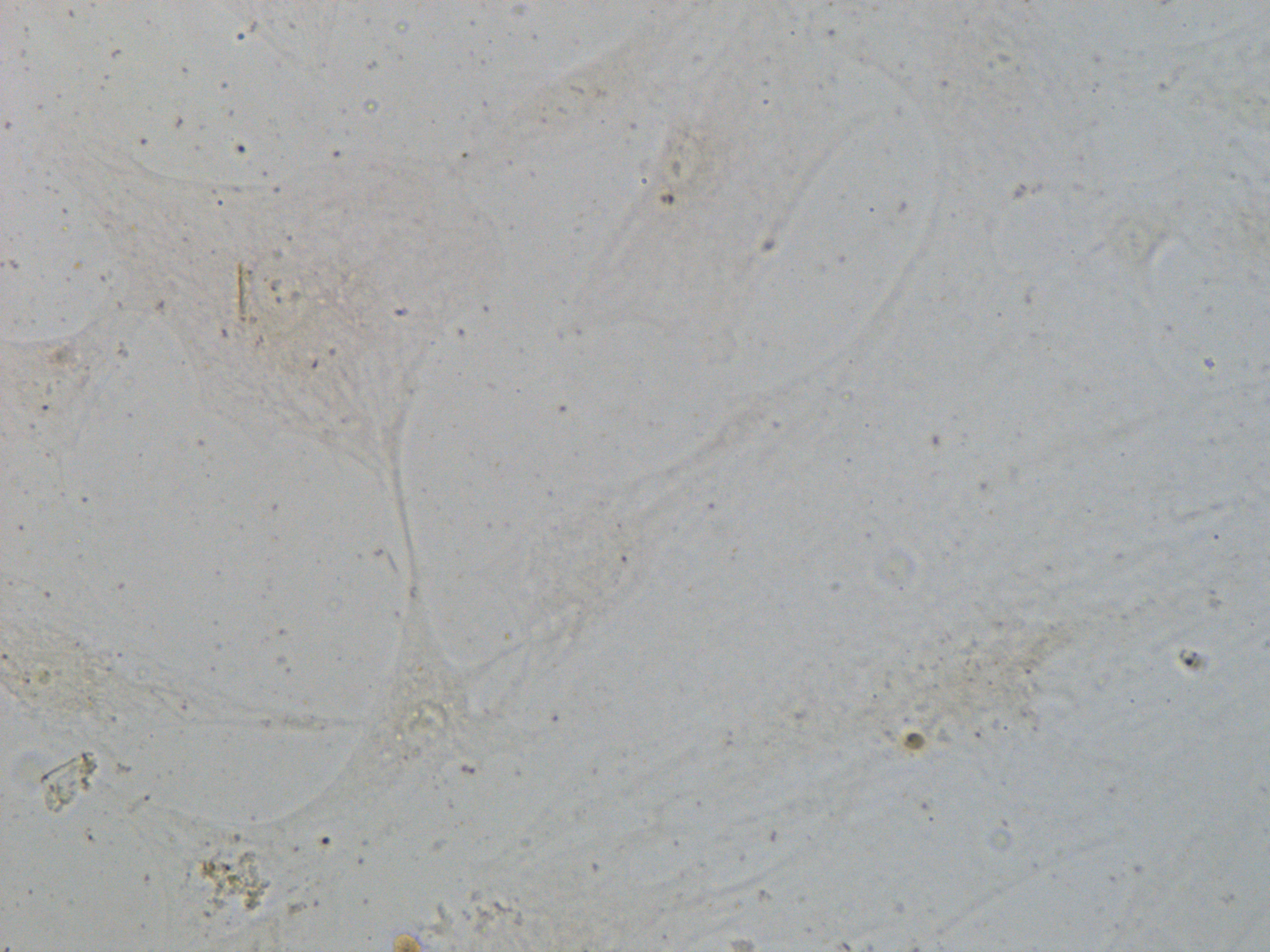

Supplement: S5 File — (ZIP) [file pone.0334482.s005.zip › Sti/ALP200-day 7-Sti-ASC10.tif]

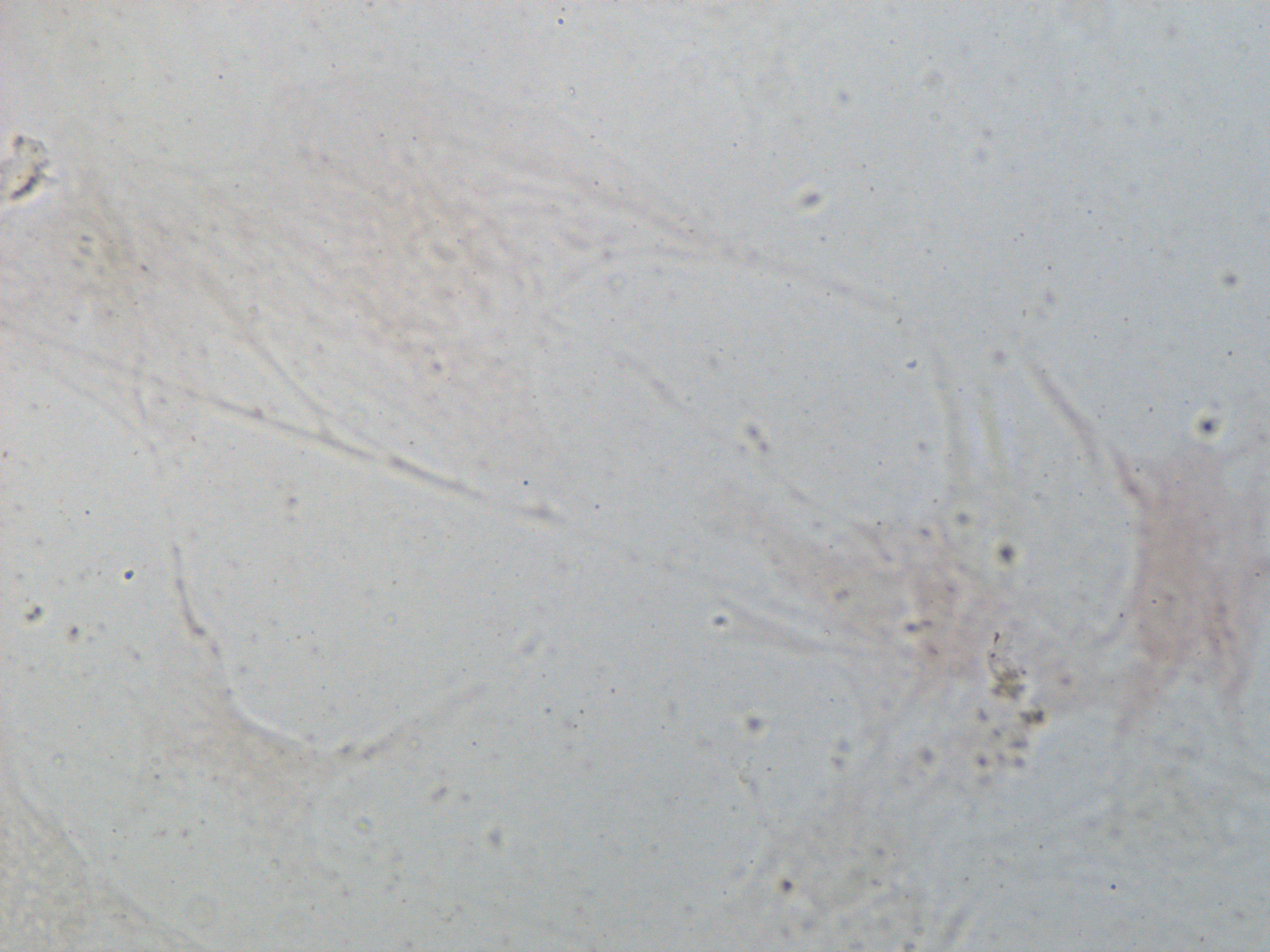

Supplement: S5 File — (ZIP) [file pone.0334482.s005.zip › Sti/ALP200-day 7-Sti-ASC8.tif]

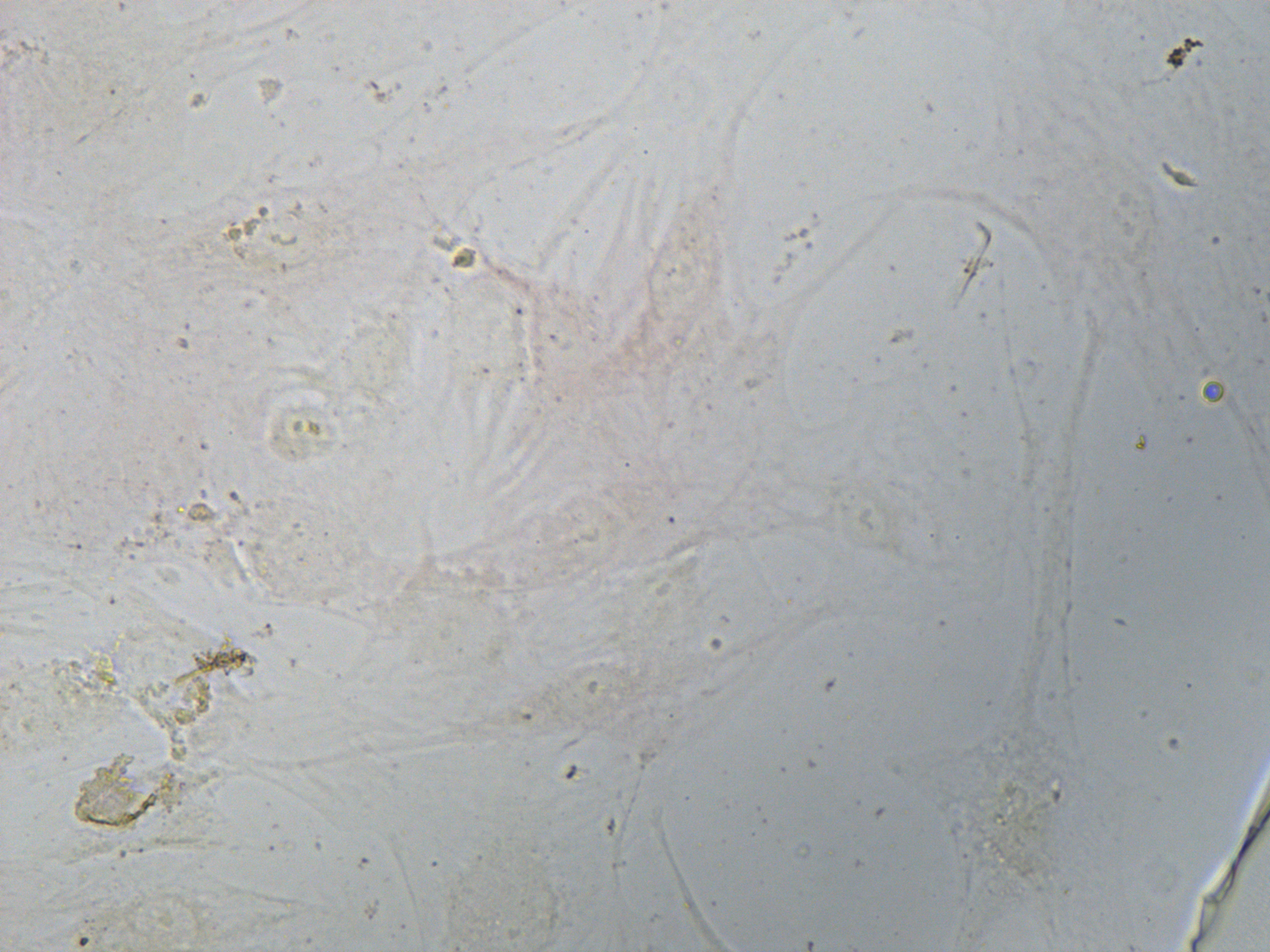

Supplement: S5 File — (ZIP) [file pone.0334482.s005.zip › Sti/ALP200-day 7-Sti-ASC9.tif]

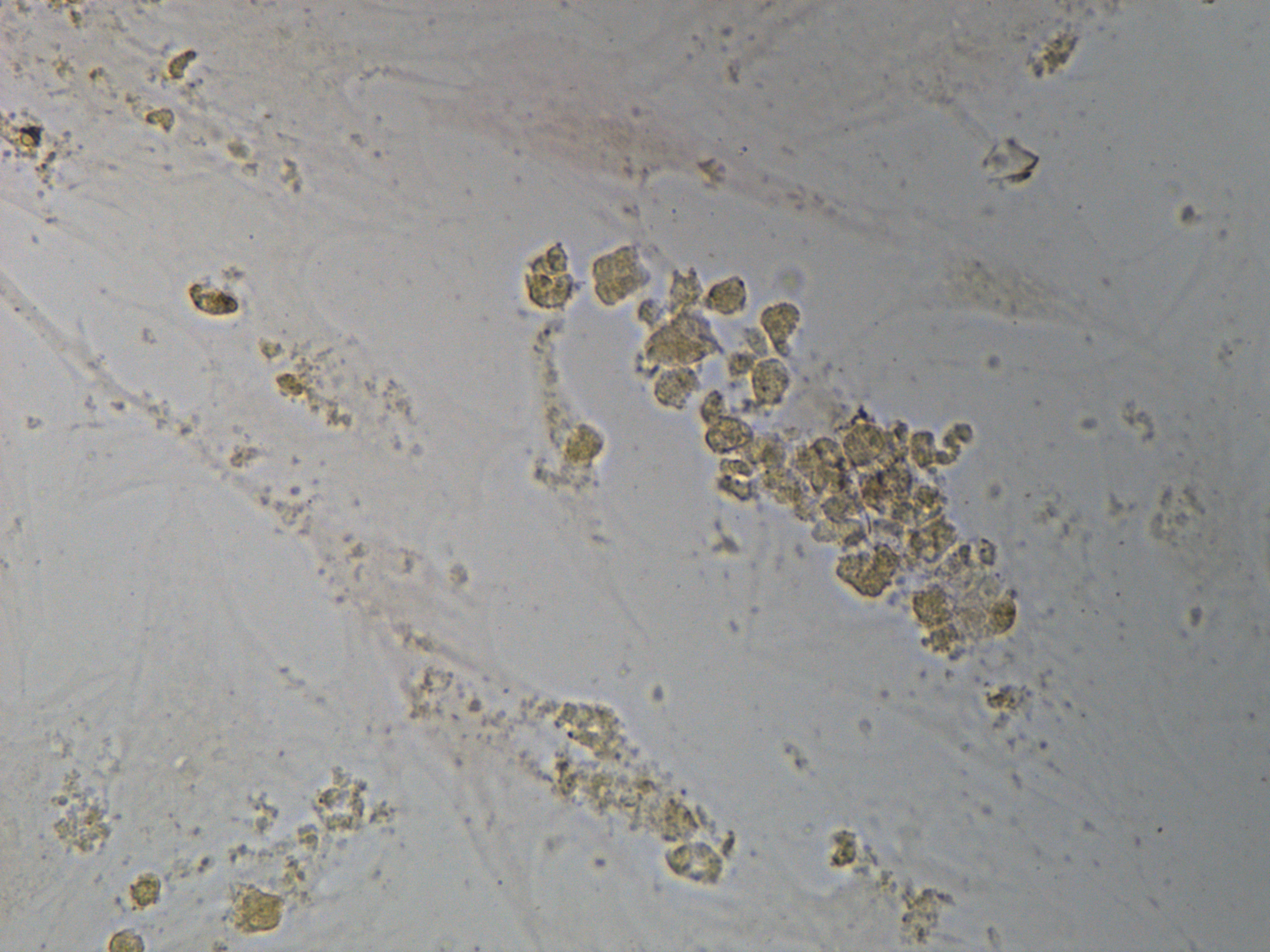

Supplement: S5 File — (ZIP) [file pone.0334482.s005.zip › Sti/ALP200-day 7-Sti-MSC11.tif]

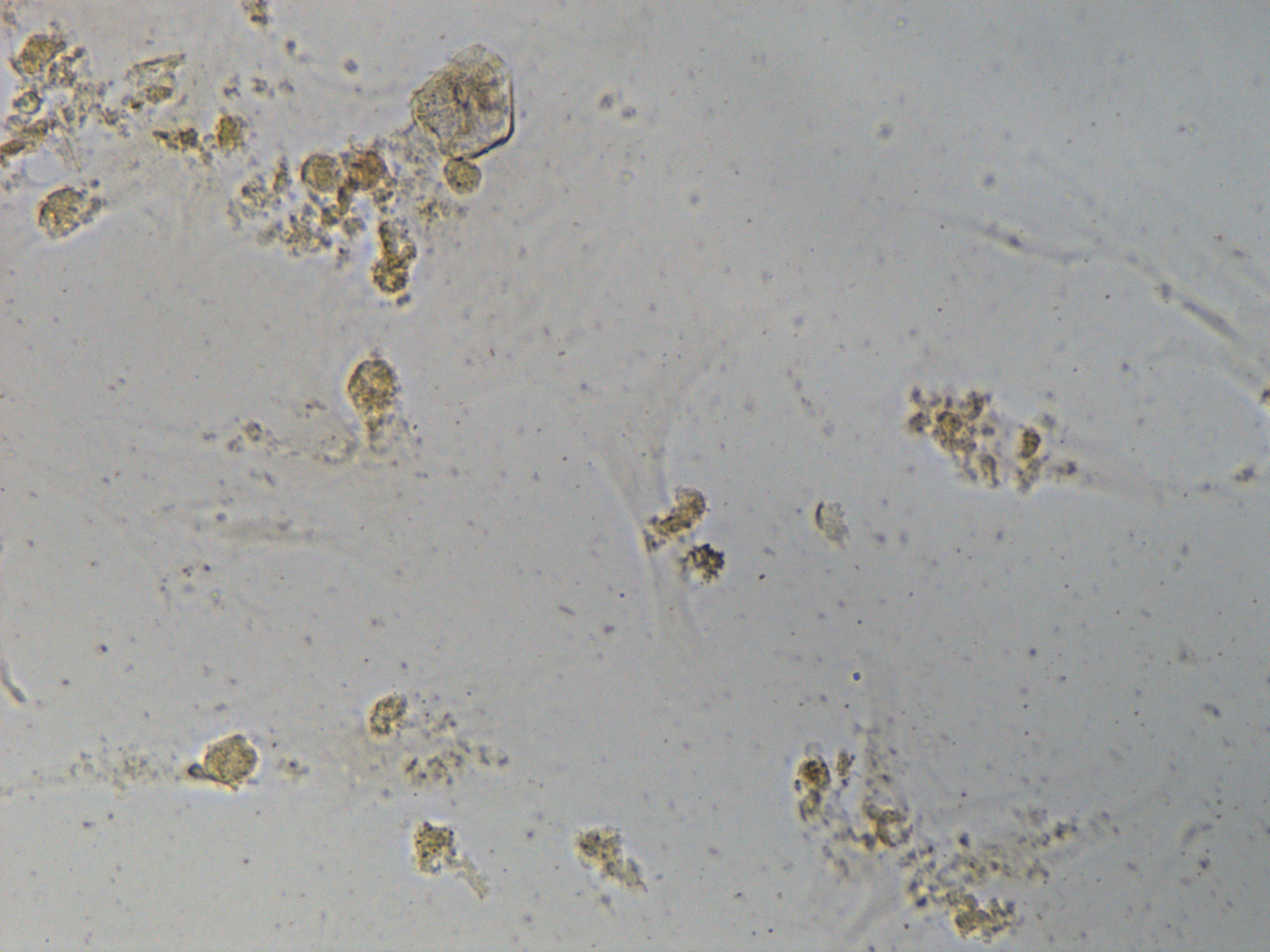

Supplement: S5 File — (ZIP) [file pone.0334482.s005.zip › Sti/ALP200-day 7-Sti-MSC5.tif]
